# Mouse‐INtraDuctal (MIND): an *in vivo* model for studying the underlying mechanisms of DCIS malignancy

**DOI:** 10.1002/path.5820

**Published:** 2021-12-13

**Authors:** Yan Hong, Darlene Limback, Hanan S Elsarraj, Haleigh Harper, Haley Haines, Hayley Hansford, Michael Ricci, Carolyn Kaufman, Emily Wedlock, Mingchu Xu, Jianhua Zhang, Lisa May, Therese Cusick, Marc Inciardi, Mark Redick, Jason Gatewood, Onalisa Winblad, Allison Aripoli, Ashley Huppe, Christa Balanoff, Jamie L Wagner, Amanda L Amin, Kelsey E Larson, Lawrence Ricci, Ossama Tawfik, Hana Razek, Ruby O Meierotto, Rashna Madan, Andrew K Godwin, Jeffrey Thompson, Susan G Hilsenbeck, Andy Futreal, Alastair Thompson, E Shelley Hwang, Fang Fan, Fariba Behbod, the Grand Challenge PRECISION Consortium

**Affiliations:** ^1^ Department of Pathology and Laboratory Medicine The University of Kansas Medical Center Kansas City KS USA; ^2^ University of Kansas School of Medicine The University of Kansas Medical Center Kansas City KS USA; ^3^ Department of Genomic Medicine The University of Texas MD Anderson Cancer Center Houston TX USA; ^4^ Department of Radiology The University of Kansas School of Medicine‐Wichita Wichita KS USA; ^5^ Department of Surgery The University of Kansas School of Medicine‐Wichita Wichita KS USA; ^6^ Department of Radiology The University of Kansas Medical Center Kansas City KS USA; ^7^ Department of General Surgery, Breast Surgical Oncology Division The University of Kansas Medical Center Kansas City KS USA; ^8^ Department of Radiology Truman Medical Center Kansas City MO USA; ^9^ Department of Pathology, St Luke's Health System of Kansas City MAWD Pathology Group Kansas City MO USA; ^10^ Heartland Pathology Wichita KS USA; ^11^ Breast Radiology Saint Luke's Cancer Institute, Saint Luke's Health System Kansas City MO USA; ^12^ Department of Biostatistics The University of Kansas Medical Center Kansas City KS USA; ^13^ Lester and Sue Smith Breast Center, Biostatistics and Informatics Shared Resources, Duncan Cancer Center Baylor College of Medicine Houston TX USA; ^14^ Department of Genomic Medicine, Division of Cancer Medicine The University of Texas MD Anderson Cancer Center Houston TX USA; ^15^ Section of Breast Surgery Baylor College of Medicine, Lester and Sue Smith Breast Center, Dan L Duncan Comprehensive Cancer Center Houston TX USA; ^16^ Duke University Department of Surgery Durham NC USA; ^17^ Department of Pathology City of Hope Medical Center Duarte CA USA

**Keywords:** ductal carcinoma *in situ*, breast cancer, nonmalignant breast cancers, Mouse‐INtraDuctal (MIND), animal models, breast malignancy, DCIS, DCIS model, precancer biology

## Abstract

Due to widespread adoption of screening mammography, there has been a significant increase in new diagnoses of ductal carcinoma *in situ* (DCIS). However, DCIS prognosis remains unclear. To address this gap, we developed an *in vivo* model, Mouse‐INtraDuctal (MIND), in which patient‐derived DCIS epithelial cells are injected intraductally and allowed to progress naturally in mice. Similar to human DCIS, the cancer cells formed *in situ* lesions inside the mouse mammary ducts and mimicked all histologic subtypes including micropapillary, papillary, cribriform, solid, and comedo. Among 37 patient samples injected into 202 xenografts, at median duration of 9 months, 20 samples (54%) injected into 95 xenografts showed *in vivo* invasive progression, while 17 (46%) samples injected into 107 xenografts remained non‐invasive. Among the 20 samples that showed invasive progression, nine samples injected into 54 xenografts exhibited a mixed pattern in which some xenografts showed invasive progression while others remained non‐invasive. Among the clinically relevant biomarkers, only elevated progesterone receptor expression in patient DCIS and the extent of *in vivo* growth in xenografts predicted an invasive outcome. The Tempus XT assay was used on 16 patient DCIS formalin‐fixed, paraffin‐embedded sections including eight DCISs that showed invasive progression, five DCISs that remained non‐invasive, and three DCISs that showed a mixed pattern in the xenografts. Analysis of the frequency of cancer‐related pathogenic mutations among the groups showed no significant differences (KW: *p* > 0.05). There were also no differences in the frequency of high, moderate, or low severity mutations (KW; *p* > 0.05). These results suggest that genetic changes in the DCIS are not the primary driver for the development of invasive disease. © 2021 The Authors. *The Journal of Pathology* published by John Wiley & Sons Ltd on behalf of The Pathological Society of Great Britain and Ireland.

## Introduction

Human ductal carcinoma *in situ* (DCIS) is increasingly diagnosed due to advances in imaging technology and an increase in routine mammographic screening [1]. At present, nearly all women diagnosed with DCIS receive aggressive therapies, including surgery, radiation, and anti‐hormonal therapy, as the standard of care. However, while most women with DCIS are treated with this clinical approach, there has not been a dramatic reduction in the incidence of invasive breast cancer, which would be expected if a precursor lesion was treated and prevented from progressing. Simulation studies comparing expected rates of DCIS progression to invasive ductal carcinoma (IDC) with data reported by the Surveillance, Epidemiology, and End Results (SEER) registry indicated that rates of DCIS‐to‐IDC transition were significantly lower than expected [1]. In 2020, in the US alone, an estimated 276 480 women were diagnosed with invasive breast cancer, along with 48 530 cases of DCIS (American Cancer Society Inc, Breast Cancer Facts and Figures; 2020). Following a DCIS diagnosis, current management reduces the risk of breast cancer recurrence, but the subsequent mortality rate (~3%) has remained unaffected. Furthermore, autopsies on middle‐aged women (40–70 years old) with no known breast disease revealed that 8.9% (0–14%) had undiagnosed DCIS lesions [2]. These data have led many researchers to believe that human DCIS is currently overdiagnosed and overtreated.

A goal of therapy for DCIS is to prevent the development of invasive breast cancer; however, the natural history of DCIS progression to invasive breast cancer is largely unknown. Several studies have investigated the risk of DCIS progressing to invasive disease by following patients who were originally misdiagnosed with benign breast diseases in whom a subsequent examination of their biopsies showed DCIS, representing an untreated model [3, 4, 5, 6]. The average rate of invasive progression for all such studies, including small case reports of patients followed for 1–30 years, was 40–50% [3, 4, 5, 6, 7]. Maxwell *et al* reported the first retrospective longitudinal study of untreated DCIS, consisting of 89 eligible women diagnosed through breast imaging and core needle biopsy between 1998 and 2010. Overall, 33% developed invasive breast cancer after a median time of 3.75 years (range 1–12 years). Among the invasive breast cancer that developed, 48% of tumors were high grade, 32% were intermediate grade, and 18% were low grade. The risk factors significantly associated with the development of invasive disease were high grade, calcifications, young age (<60 years), and the absence of anti‐hormonal therapy [6]. Furthermore, among women with low‐grade DCIS, survival was similar with or without surgery (follow‐up of ~6 years) [8]. These data have provided the basis for clinical trials evaluating the safety of ‘active surveillance’ for low‐grade DCIS, including LORIS [9], COMET [10], LORD [11], and LORETTA [12].

Despite previous studies, there is still no clear signature to predict the future invasive potential of DCIS, in part reflecting the scientific challenge posed by the diversity of human DCIS. One limitation in prior studies was the lack of reliable *in vivo* models for basic and translational research. To address this gap, we present a patient‐derived xenograft (PDX) model of DCIS, referred to as Mouse‐INtraDuctal (MIND), in which patient DCIS epithelial cells are injected intraductally and studied as they progress naturally *in vivo*. Our group originally reported the use of MIND models for studying DCIS progression using established cell lines [13]. The DCIS‐like lesions generated from MCF10DCIS.com and SUM225CWN cell lines formed after 2 weeks and slowly progressed to invasive lesions in 10–14 weeks [13]. We also reported the reproducible growth of patient DCIS epithelial cells in NOD‐SCID IL2rγ (ΝSG) mice using the MIND method. However, at that time, the models were followed for only 8 weeks, by which time none of the lesions showed invasive progression or recapitulated patient histologic or pathologic characteristics. We now report the development of a larger number of patient‐derived xenografts with a median follow‐up of 9 months in which only a fraction (54%) developed invasive lesions, while the remaining (46%) remained non‐invasive. As such, the models provide a valuable tool to elucidate the epithelial inherent and patient specific mechanisms that underlie DCIS invasiveness. Additionally, the models provide a valuable resource for the development of therapeutic strategies for prevention of DCIS malignancy.

## Materials and methods

### Specimen collection

Patients gave informed consent to participate in this University of Kansas Medical Center Institutional IRB approved study. Upon consent, an extra core biopsy or surgical specimen was obtained for research. Recruited subjects included patients undergoing image‐guided core needle biopsy or surgical excision (lumpectomy or mastectomy) due to suspected DCIS. In all cases, research specimens were obtained only after the acquisition of diagnostic specimens. Following collection, biopsy tissue was placed in preservation media (LiforCell, Lifeblood Medical, Inc, Adelphia, NJ, USA) and stored at 4 °C or on ice until processing to isolate epithelial and stromal cell components.

### Tissue digestion

Upon the receipt of DCIS tissue samples, the tissue was weighed and then transferred to a Teflon block, finely minced with scalpels, and transferred to a 50‐ml conical tube containing freshly prepared, filter‐sterilized digestion medium [10 ml per g of tissue; contained 5 mg of collagenase (Roche Applied Science, Indianapolis, IN, USA), 0.24 mg of hyaluronidase (2140 units/mg; Sigma‐Aldrich, St Louis, MO, USA), 200 mg of BSA, 100 μl of antibiotic‐antimycotic (Thermo Fisher Scientific, Waltham, MA, USA), and 10 ml of DMEM/F12]. Following incubation with rotation for 16 h (50 rpm at 37 °C), the specimens were removed, briefly shaken by hand, and centrifuged at 200 × *g* for 1 min. Prewarmed trypsin–EDTA (1 ml; Stem Cell Technologies, Vancouver, BC, Canada) was added to the resulting pellet and gently pipetted up and down for 1 min. Hank's balanced salt solution (HBSS) with 2% fetal bovine serum (FBS) (HF) was added, and specimens were centrifuged at 400 × *g* for 5 min. The supernatant was removed, and 1 ml of prewarmed 5 mg/ml Dispase (Stem Cell Technologies) and 100 μl of 1 mg/ml DNase I (Stem Cell Technologies) were added to the pellet. To resuspend the pellet, the specimen was pipetted up and down for 1 min. An additional 10 ml of cold HF was added to the cell suspension, which was filtered through a 40‐μm cell strainer. The cell suspension was centrifuged at 400 × *g* for 5 min; the resulting cell pellet was resuspended in 200 μl of phosphate‐buffered saline (PBS) and cells were counted. Cells were then frozen in 93% FBS and 7% DMSO and stored in liquid nitrogen until intraductal injection.

### Animals and MIND surgeries

Recipient mice were 8‐ to 10‐week‐old virgin female NOD‐SCID IL2Rgamma‐null (NSG) mice, which were purchased from The Jackson Laboratory (Bar Harbor, ME, USA). Animal experiments were conducted following protocols approved by the University of Kansas School of Medicine Animal Care and Use and Human Subjects Committee.

For MIND surgeries, a Hamilton syringe with a 50‐μl capacity and a blunt‐ended ½'' 30‐gauge needle was used to deliver the cells as described previously [13]. Two microliters of PBS (with 0.04% trypan blue) containing ~35 000 cells were injected. After 6–12 months, mice were sacrificed, and mammary tissues were fixed and processed for embedding.

To measure the extent of growth, mammary glands containing xenografted lesions were fixed overnight in 4% paraformaldehyde and processed into paraffin wax. Each entire gland was serially sectioned at 5 μm. Every tenth section was mounted, and human cells growing in the mouse gland were identified by immunofluorescence (IF) using a human specific anti‐CK19 antibody following epitope retrieval in a high‐pH Tris buffer. Growth areas on every tenth section were imaged at 20× magnification on a Zeiss Imager M‐2 microscope (Zeiss, Oberkochen, Germany) fitted with an AxioCam MRm camera (Zeiss). The perimeter of all growth areas was drawn using the outline tool in Axiovision software (Zeiss), and the area of growth was measured in μm^2^. Total growth was determined by adding the positive areas from all imaged slides.

### Immunohistochemistry (IHC)

Antibodies used for IF and IHC are listed in supplementary material, Table S1. Paraffin sections cut at 5 μm were mounted on Fisherbrand Superfrost slides (Thermo Fisher Scientific, Pittsburgh, PA, USA), incubated for 60 min at 60 °C, and then deparaffinized and rehydrated. Epitope retrieval was performed in a Decloaking Chamber (Biocare Medical, Pacheco, CA, USA) under pressure for 5 min using citrate buffer, pH 6.0, followed by a 10‐min cooling down period. Endogenous peroxidase was blocked using 3% H_2_O_2_ for 10 min, followed by incubation with primary antibody for 40 min followed by Mach 2 HRP‐Polymer (BioCare, Concord, CA, USA) for 30 min, and 3,3'‐diaminobenzidine (DAB)+chromogen (Dako, Carpinteria, CA, USA) for 5 min. Immunohistochemical staining was performed using an IntelliPATH FLX Automated Stainer (Biocare Medical, Pacheco, CA, USA) at room temperature. Light hematoxylin counterstaining was performed, after which the slides were dehydrated, cleared, and mounted using a permanent mounting medium.

### Immunofluorescence (IF)

IF was performed as previously described [14]. Antibodies are listed in supplementary material, Table S1. Nuclei were counterstained with Hoechst (Thermo Fisher, Grand Island, NY, USA). Negative controls were carried out using secondary antibodies without primary antibodies. Imaging was performed using a laser‐scanning confocal microscope (Model 510; Carl Zeiss MicroImaging, Inc, Thornwood, NY, USA). The acquisition software used was Pascal (Carl Zeiss MicroImaging, Inc). Fluorescence quantitation and analysis were done using MetaMorph® Microscopy Automation and Image Analysis Software (Molecular Devices, San José, CA, USA).

### Mouse mammary gland processing and magnetic sorting

To allow re‐transplantation of MIND DCIS cells, mammary glands from MIND xenografts were excised (at 12 months after intraductal injection) and digested overnight as described above. Single mammary epithelial cells were then magnetically labeled with mouse MHCI/II antibodies (listed in supplementary material, Table S1) and with MACS Anti‐Biotin MicroBeads UltraPure (#130‐105‐637; Miltenyi Biotec, Bergisch Gladbach, Germany), and negatively sorted for human DCIS cells using Miltenyi LD columns (#130‐042‐901; Miltenyi Biotec) following the manufacturer's protocol. A sample of sorted cells was then analyzed by fluorescence‐activated cell sorting (FACS) to examine the purity, and the rest of the cells were injected at 25,000 cells per mammary gland.

### Flow cytometry

Cells were stained at a final antibody dilution of 1:100 for 30 min on ice followed by washes in HBSS (#24020‐117; Invitrogen, Grand Island, NY, USA) containing 2% FBS. The antibodies used are listed in supplementary material, Table S1. FACS and data analysis were performed using a BD LSR II flow cytometer (BD Pharmingen, Franklin Lakes, NJ, USA) and FlowJo software (Tree Star, Ashland, OR, USA).

### Laser capture microdissection (LCM) and DNA sequencing

Formalin‐fixed, paraffin‐embedded (FFPE) specimens were cut at 10 μm, placed on slides, and stored overnight to air dry. The slides were then deparaffinized and stained using a Paradise staining kit (Arcturus™ Paradise™) (#KIT0312S; Thermo Fisher Scientific) in preparation for microdissection. Microdissection was performed using an ArcturusXT™ LCM System (Thermo Fisher Scientific) following the system manual. A PicoPure™ DNA Extraction Kit (#KIT0103; Thermo Fisher Scientific) was used for DNA extraction, and DNA quantification was performed fluorimetrically (Qubit 2.0, #Q32866; Invitrogen).

#### DNA sequencing

To compare molecular aberrations between patient DCIS and corresponding xenografts, a high‐depth targeted sequencing platform of 201 genes was utilized [15]. Libraries were made from 100–200 ng of DNA extracted from LCM‐captured FFPE sections of patient DCIS, adjacent normal tissues, and corresponding DCIS xenografts. A total of 201 genes were captured, and sequencing was performed using an Illumina HiSeq2000 (Illumina Inc, San Diego, CA, USA). Duplicate reads were removed from the raw data, and the reads were mapped to the hg19 reference genome. This study enabled the detection of very low frequency mutations (as low as 5% and higher).

#### Sequencing data processing and variant filtering

Sequencing data were converted to a FASTQ format and then aligned to the hg19 reference genome using the Burroughs–Wheeler Aligner (BWA) [16]. The aligned BAM files were subjected to mark duplication, realignment, and recalibration using Picard (www.broadinstitute.org/picard) and GATK (www.broadinstitute.org/gatk). The BAM files were then analyzed by MuTect2 (http://gatk.broadinstitute.org) and Pindel [17] against the normal sample of the individual to detect somatic SNVs and insertions/deletions (indels), respectively. Variants were further filtered by the following criteria to investigate shared variants among the samples: (1) variants in genes of the T200.1 panel; (2) log odds score ≥ 10; (3) exonic variants; (4) the variant site was covered by at least 1 read in both the tumor and the normal samples.

#### Quality control

Individuals with tumor sample median target coverage < 50 were excluded from further analysis.

### Tempus XT assay

#### Data analysis and the generation of a heatmap

Genes with mutations in at least two individuals were plotted in a heatmap. Both the columns and the rows were ordered by hierarchical clustering. Variant severity was called using SnpSift, which is a program for identifying candidate phenotype‐relevant variants [18, 19].

##### Sequencing data processing and variant filtering

The tumor‐only samples were sequenced by Tempus Labs (Chicago, IL, USA) using the Tempus|xT 648‐gene panel. Tempus utilizes an in‐house CAP‐accredited, CLIA‐certified robotic sequencing lab for sequencing, with automated bioinformatics and variant classification reporting. Exonic variants or intronic variants that affect the splice region were further prioritized for downstream analysis. The heatmap was plotted using the R package ‘ComplexHeatmap’ [20].

### Statistical analysis

All statistical analysis was performed using R Statistical Software (version 1.3.1056; R Foundation for Statistical Computing, Vienna, Austria). A Kruskal–Wallis chi‐squared test was used to compare statistical differences in extent of growth among the xenografts.

Logistic regression was used for analyzing whether any biomarkers predicted invasive progression.

A Kruskal–Wallis chi‐squared test was used also for calculating the frequency of mutations in each group. ANOVA was used for comparing differences in the duration of follow‐up for the xenografted DCIS in each group.

## Results

### Mouse‐INtraDuctal (MIND) represents the first *in vivo* model to recapitulate the entire spectrum of human DCIS pathology

**Figure 1:**
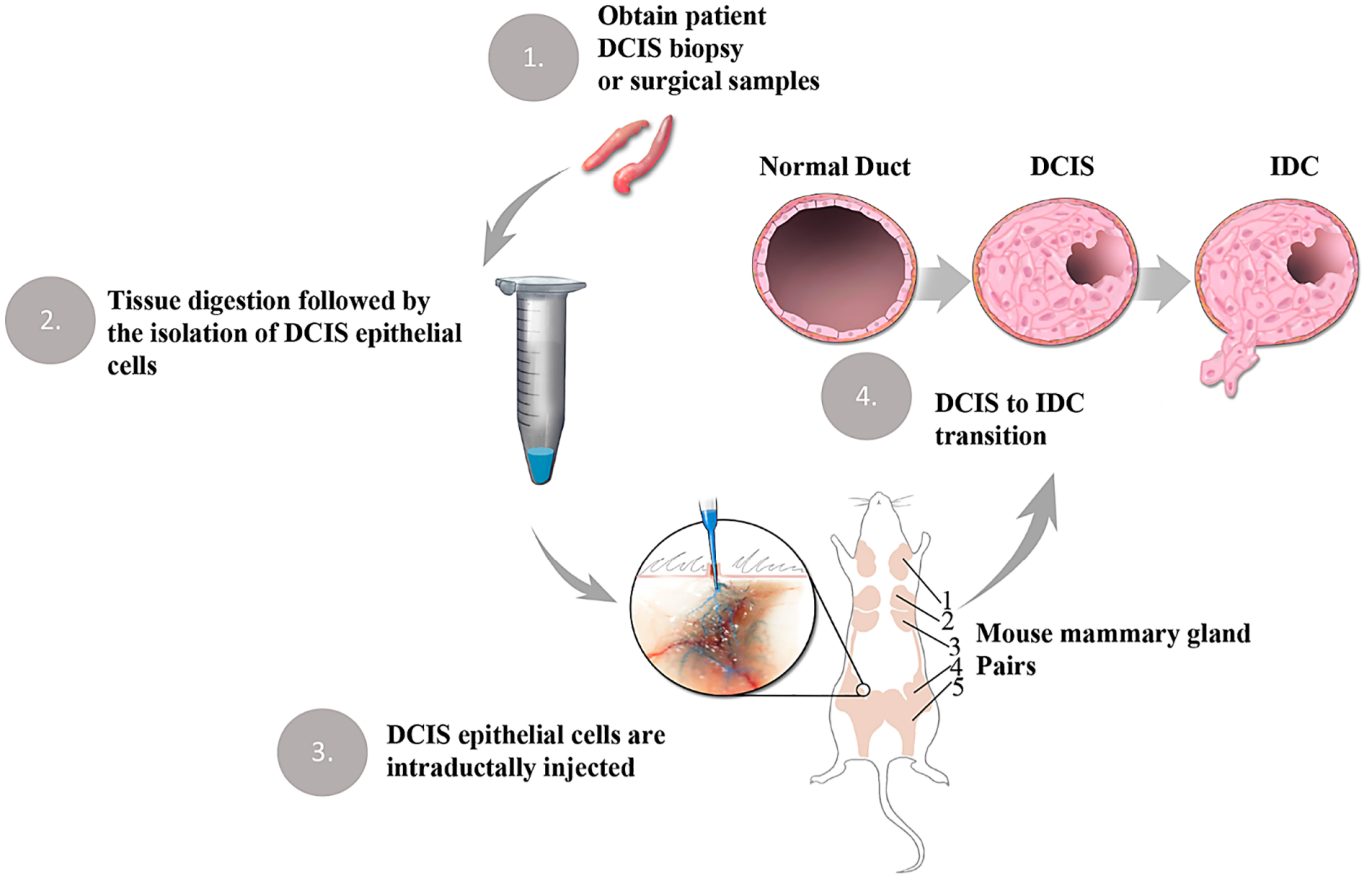
Mouse INtraDuctal (MIND) model. MIND involves the intraductal injection of patient‐derived DCIS epithelial cells into the mammary ducts of immunocompromised mice. (1–2) DCIS epithelial cells are obtained following an overnight digestion of patient DCIS biopsy or surgical samples. (3) DCIS cells are injected into the primary mouse mammary ducts via the nipple. (4) Engrafted epithelial cells form *in situ* lesions and a fraction becomes invasive by bypassing the myoepithelial layer and the basement membrane. The brown regions in the outline of the mouse are mammary gland pairs 1–5.

DCIS progression has been difficult to study due to the paucity of useful animal models. To address this deficiency, we developed an *in vivo* DCIS progression model, Mouse‐INtraDuctal (MIND), in which human DCIS epithelial cells or DCIS cell lines are injected intraductally and studied over time in immunocompromised mice (Figure 1).

Between 2009 and 2019, we collected over 1100 surgical and biopsy specimens that were initially suspected of DCIS based on radiologic examination. Subsequent pathologic examination confirmed DCIS or other pathologies. Of the 375 cases that received a final diagnosis of pure DCIS (supplementary material, Figure S1A), the samples represented different molecular subtypes based on estrogen receptor (ER), progesterone receptor (PR), and HER2 expression (supplementary material, Figure S1B). Intraductal injection of epithelial cells derived from various breast pathologies including DCIS showed >70% rate of engraftment (supplementary material, Figure S1C–E).

**Figure 2:**
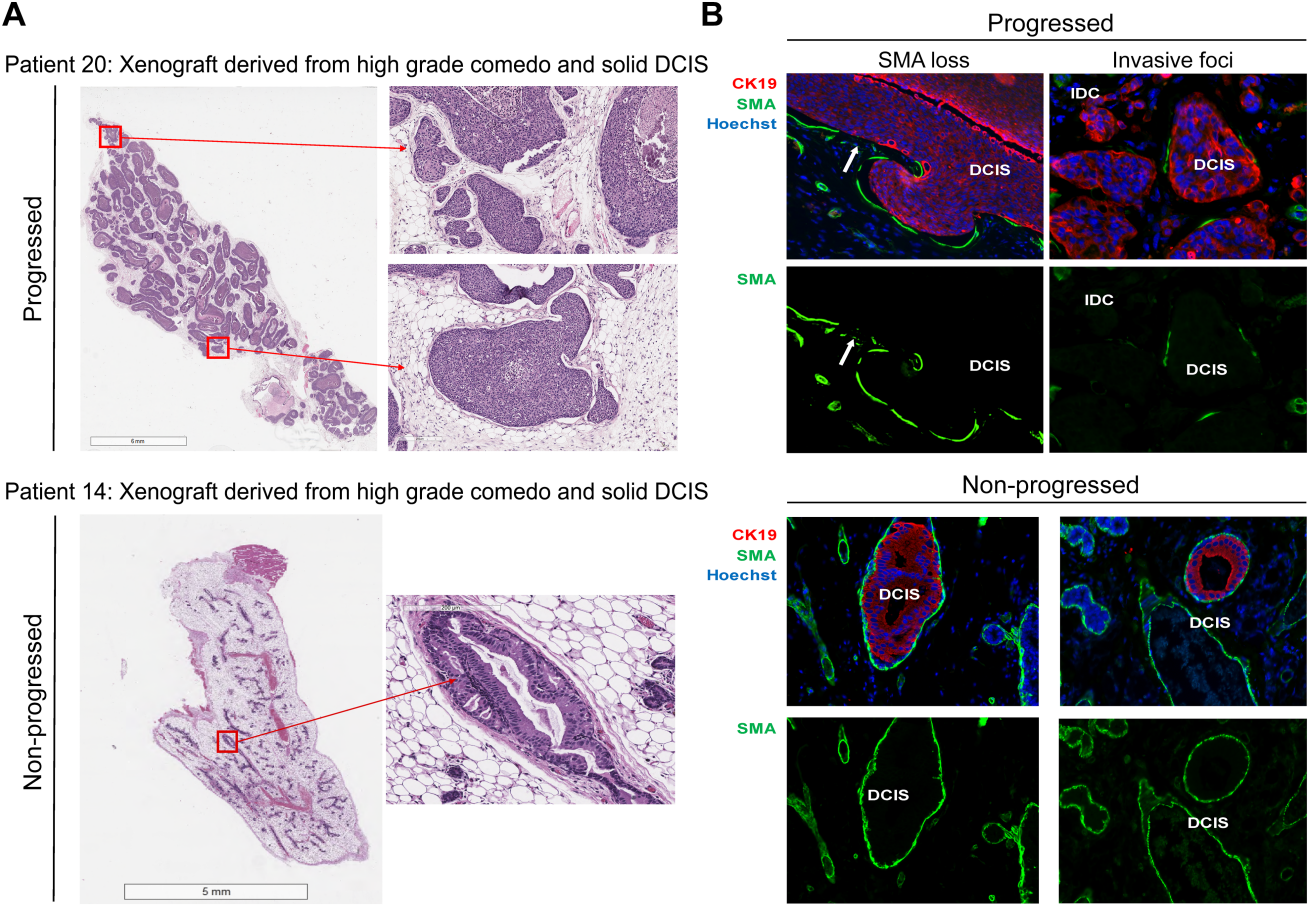
MIND supports the natural evolution of human DCIS in mice. (A) Representative images of a progressed DCIS xenograft (patient 20) and a non‐progressed DCIS xenograft (patient 14). Panel A shows whole‐gland cross‐section images (left) and magnified views (right). (B) Representative images of human‐specific CK19 immunofluorescence (red), SMA (green), and Hoechst nuclear dye (blue) demonstrating loss of the myoepithelial layer around the progressed lesions, while the SMA layer remained intact around the non‐progressed lesions. Arrows point to intraductal lesions that lost SMA.

To assess DCIS progression in the MIND models, immunofluorescence (IF) staining was performed on sections of mammary glands injected intraductally with patient‐derived DCIS epithelial cells. Anti‐smooth muscle actin (SMA) antibody was used to identify myoepithelial cells, and human‐specific anti‐cytokeratin (CK) 19 to identify human epithelial cells. We evaluated invasive progression and microinvasion by the loss of SMA surrounding the xenografted DCIS lesions on three consecutive FFPE sections, as this was indicative of compromised myoepithelium [21]. Although the loss of SMA was often noted in areas of significant growth, some SMA loss could also be observed in lesions that were only a single‐cell layer thick, suggesting possible interactions between xenograft DCIS epithelial cells and the mouse myoepithelial layer that was not purely mechanical. We previously demonstrated using IF *in situ* hybridization (IFISH) that the SMA layer was composed of mouse cells rather than human cells [13]. Figure 2 shows an example of a progressed (top panel) versus a non‐progressed (bottom panel) DCIS xenograft. The progressed xenograft was generated by the intraductal injection of DCIS epithelial cells from a patient sample that showed comedo and solid histology. As shown in the hematoxylin and eosin (H&E) images (Figure 2A, top left), the DCIS xenograft exhibited ductal filling, extensive growth, and the loss of an intact SMA layer (Figure 2B, top right). In addition, similarly to the patient DCIS lesion, the progressed xenograft formed solid and comedo lesions that extended throughout the entire mouse mammary fat pad. The non‐progressed lesion was generated by the intraductal injection of DCIS epithelial cells from a patient with cribriform DCIS. This xenograft exhibited minimal growth that contained single‐ and multi‐layered epithelium that retained an intact SMA layer (Figure 2A,B, bottom panels). DCIS in patient 20 was ER^−^/PR^−^, while DCIS in patient 14 was ER^+^/PR^+^. Both patients' DCISs were of high nuclear and histologic grades.

Additional representative IF images of xenografted DCIS lesions from progressed and non‐progressed xenografts are presented in supplementary material, Figure S2, which shows that the non‐progressed DCIS‐like lesions in the MIND models were surrounded by an intact myoepithelial layer, while the progressed models showed either a total loss or a discontinuous myoepithelial layer.

**Figure 3:**
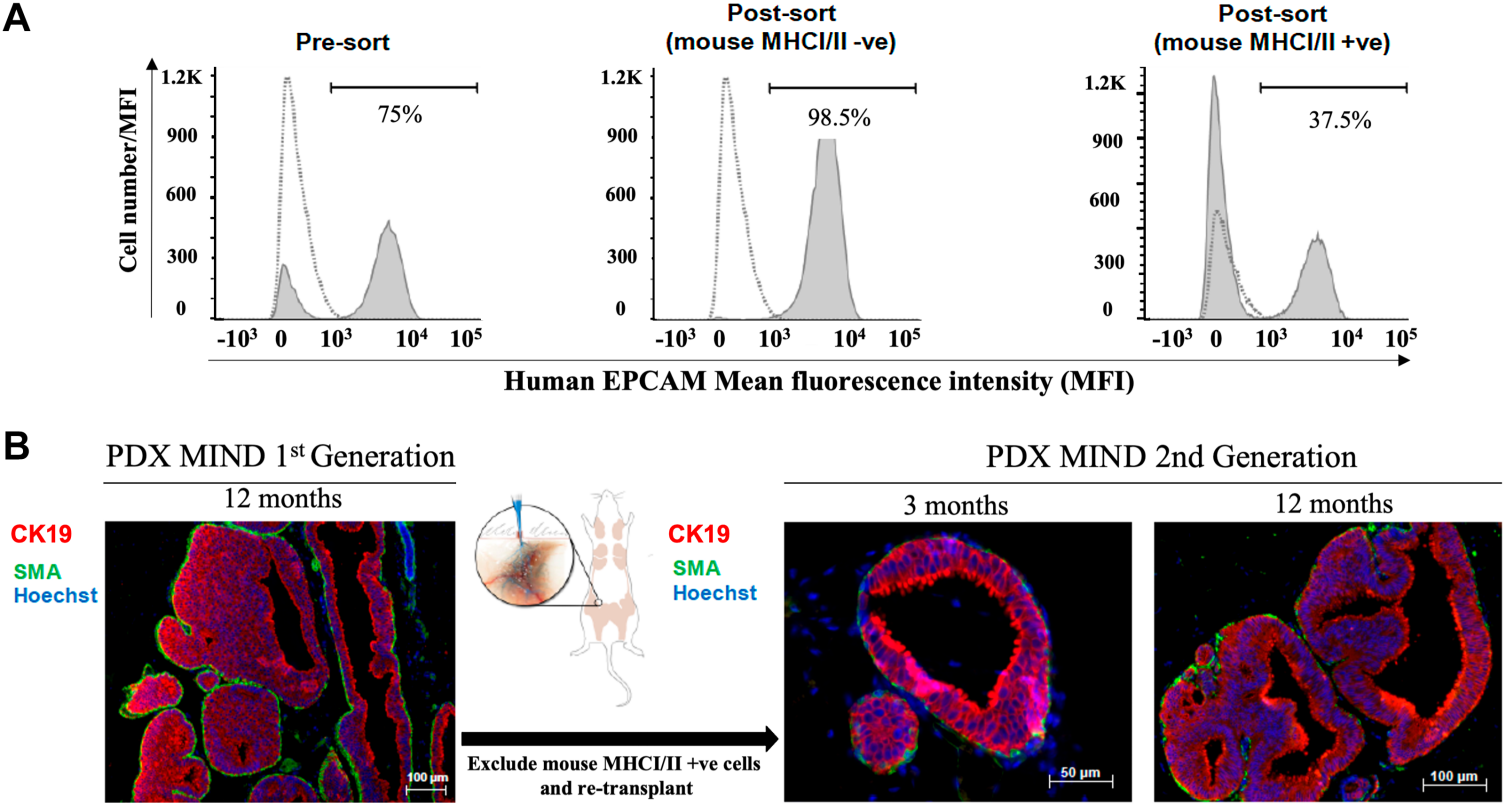
DCIS MIND xenografts retain their histologic features with sequential transplantation. DCIS epithelial cells were sorted magnetically and sequentially transplanted into a second generation of mice. (A) Flow cytometry analysis showing the proportion of EpCAM‐positive cells before sorting (pre‐sort), following the exclusion of mouse cells using anti‐mouse MHC I/II (post‐sort), and in the mouse cell subpopulation positively sorted using anti‐mouse MHC I/II. The dashed lines represent an isotype control that is overlaid on the pre‐ and post‐sort graphs. One isotype control was used to overlay on both pre‐ and post‐sort graphs. (B) Representative immunofluorescence images of first generation (12 months) and second generation (3 and 12 months) following transplantation. Anti‐human CK19 (red), anti‐SMA (green), and Hoechst nuclear dye (blue).

For one case, DCIS cells from the first‐generation transplants were passaged into a second generation. After 12 months in the first‐generation xenograft, human DCIS epithelial cells were magnetically sorted by exclusion of mouse cells using antibodies to mouse MHC I/II, followed by transplantation into second‐generation xenografts. The negatively sorted human epithelial cells were mainly human EPCAM‐positive cells (98.5%) (Figure 3A). As shown in Figure 3B, xenografted second‐generation DCIS preserved the original patient's histologic features (patient 23), cribriform, at 3 and 12 months following transplantation.

A comprehensive list of available data for the patients and xenografts is provided in supplementary material, Table S2. The data include patient and xenograft IDs, progression status for the xenografts, extent of *in vivo* growth (median ± IQR) for the xenografts, level of biomarker expression for the xenografts and patient DCIS, tumor and nuclear grades, and histology for the xenografts and patient DCIS as well as patient demographics. Among the 37 patient samples injected by the MIND method and followed for a median duration of 9 months, 20 samples (54%) injected into 95 xenografts showed *in vivo* invasive progression, while 17 (46%) samples injected into 107 xenografts remained non‐invasive. Among the progressed xenografts, nine patient samples injected into 54 xenografts exhibited a mixed pattern in which some xenografts showed invasive progression while others remained non‐invasive. Notably, due to heterogeneity in growth patterns and the success of intraductal injections, each #4 mammary gland was counted as one individual xenograft and labeled accordingly. Although, intraductal injections were performed into #4 mammary glands on both sides, occasionally injection to the left gland or right gland failed. In those cases, we evaluated only the gland in which the intraductal injection was successful. The success of intraductal injection was assessed by the spread of trypan blue into the entire mammary gland. Therefore, it would have been unfair to count one gland as one mouse in which only the intraductal injection to one gland was successful versus two glands as one mouse in which intraductal injections to both glands were successful. In addition, we noticed significant variability in the extent of DCIS growth for each gland of the same mouse. In some cases, there was minimal growth on one side while there was extensive growth on the other side. Therefore, we decided to count each gland as *n* = 1 or as one xenograft and report the results accordingly. The mean duration of follow‐up was not significantly different between the progressed versus non‐progressed groups (non‐progressed: mean 8.82 ± 3.18 months versus progressed: mean 9.5 ± 2.4 months; mixed: mean 9.1 ± 3.12 months; one‐way ANOVA; *p* = 0.44).

### A significant association was found between PDX DCIS MIND extent of *in vivo* growth and invasive potential

**Figure 4:**
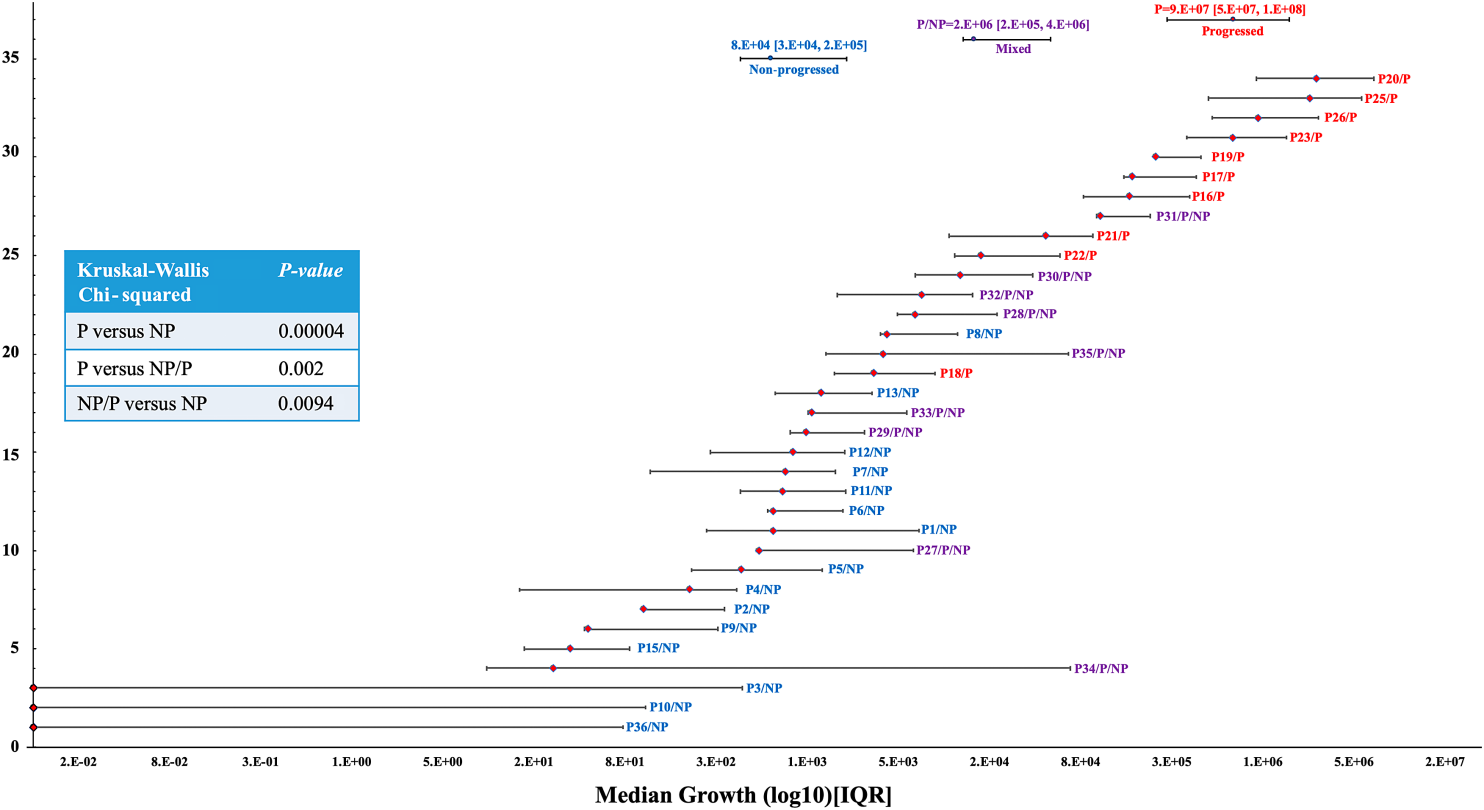
Extent of PDX DCIS *in vivo* growth showed a significant correlation with invasive progression. Forest plot of the extent of xenografted DCIS *in vivo* growth for 34 patient samples (202 xenografts). Each line represents median growth and interquartile ranges (25–75%) for PDXs derived from one patient sample. Red labels represent progressed, blue non‐progressed, and purple mixed progressed and non‐progressed samples. The inset shows the results of simple linear regression analysis, comparing the extent of DCIS *in vivo* growth among the three groups. P = progressed; NP = non‐progressed; NP/P = mixed.

To measure the extent of PDX DCIS *in vivo* growth, mammary glands containing xenografted lesions were serially sectioned (the entire gland, up to 100 sections) and stained by IF using human‐specific antibodies against CK19. Growth areas on every tenth section were combined and reported for each xenograft. As illustrated in the forest plot in Figure 4, there was a significant level of inter‐ and intra‐tumoral heterogeneity in the PDXs' extent of *in vivo* growth. Those PDXs that showed *in vivo* invasive progression were labeled as P (progressed) and those that remained non‐invasive as NP (non‐progressed). Nine patient samples exhibited a mixed progression status in which some of the PDXs showed invasive progression while others remained non‐invasive. These data demonstrate that patient samples which generated mixed models contained both invasive and non‐invasive cells. Nonetheless, Kruskal–Wallis analysis showed that the extent of PDX DCIS *in vivo* growth was significantly greater in progressed than in mixed and non‐progressed models (*p* < 0.05) (Figure 4).

### PR expression levels predicted MIND DCIS invasive potential

**Figure 5:**
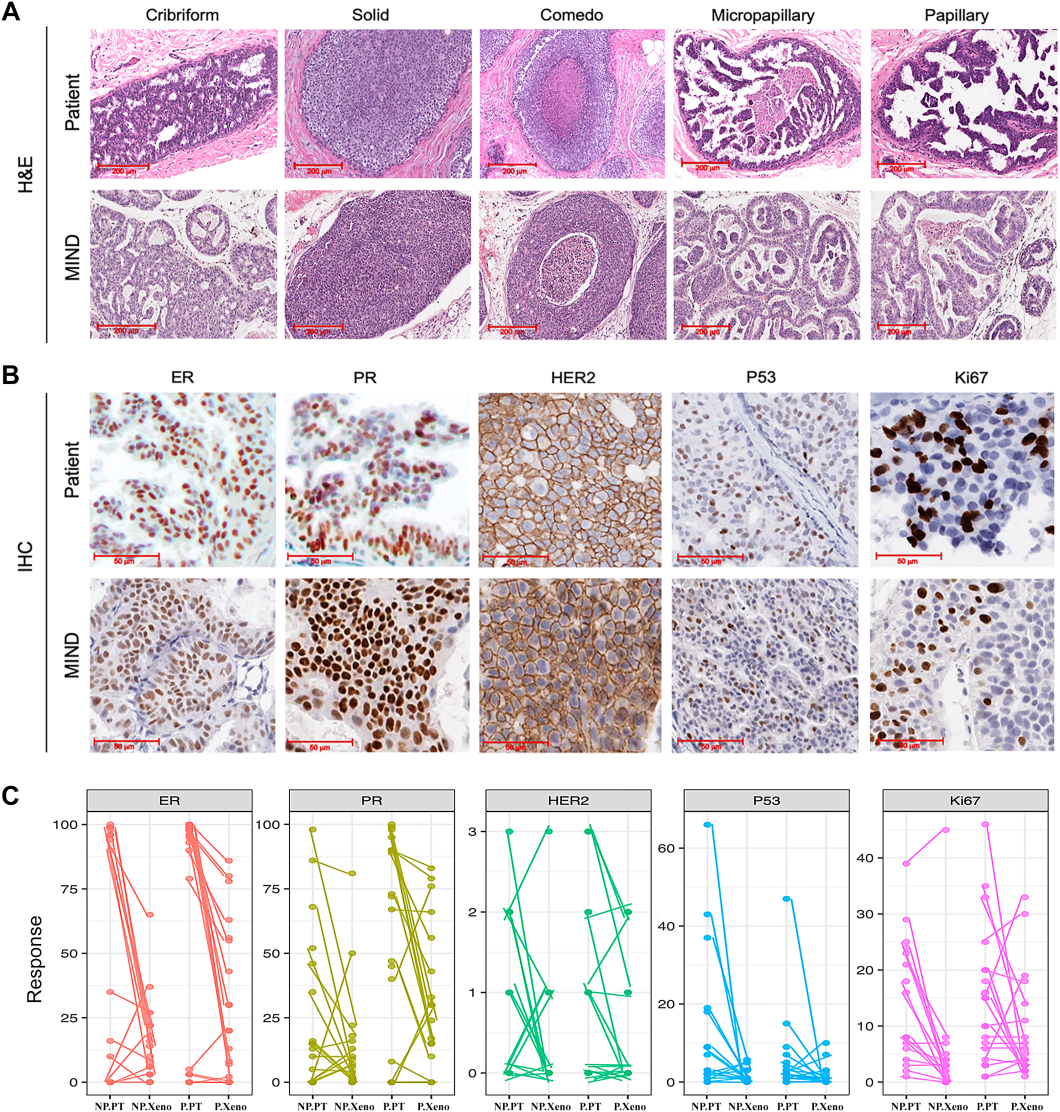
A side‐by‐side comparison of biomarkers and histology in patient DCISs and their corresponding xenografts. (A) H&E images demonstrated that xenografted DCISs support the formation of all five DCIS histologic features: cribriform, solid, comedo, micropapillary, and papillary. (B) Representative IHC images comparing patient lesions and their corresponding xenografts, demonstrating that a large proportion of MIND models retain biomarkers expressed in patient DCISs, including ER, PR, HER2, P53, and Ki67. (C) A pairwise comparison of biomarker expression in patient DCISs and their corresponding xenografts. NP.PT = non‐progressed patient; NP.Xeno = non‐progressed xenograft; P.PT = progressed patient; P.Xeno = progressed xenograft.

To address whether DCIS xenografts mimicked patient biomarkers and/or histologic features, a side‐by‐side comparison was made. Figure 5A,B shows examples of H&E staining and immunohistochemistry (IHC) for selected patient DCISs and their corresponding xenografts. As shown, patient DCIS lesions and their corresponding xenografts expressed similar biomarkers, ER, PR, HER2, Ki67, and p53 (Figure 5A,B). H&E staining demonstrated that the xenografted DCIS supported the growth of all five distinct histologic features, including cribriform, solid, comedo, micropapillary, and papillary. Figure 5C shows a pairwise comparison of biomarkers in patient DCISs and their corresponding xenografts. The guidelines established by the College of American Pathology (CAP) were followed for calling biomarker positivity for ER, PR, HER2, Ki67, and P53. Based on the guidelines, DCIS was called ER‐ and/or PR‐positive if protein expression by IHC was expressed in ≥1% of cells; HER2 < 2+ was negative, 2+ was equivocal, and 3+ was positive; the biomarkers Ki67 and p53 were referred to as positive if expressed by ≥10% of cells. We then compared biomarker positivity on patient sections versus xenograft sections. Biomarkers were called discordant if the patient sample and the corresponding xenograft switched from positive to negative and vice versa. For example, if patient DCIS was ER 2% (positive) and xenograft ER was 10% (positive), ER expression on patient and xenograft was referred to as concordant. However, if patient DCIS was ER 2% (positive) and the xenograft ER expression was 0% (negative), ER expression was referred to as discordant. As shown in supplementary material, Table S3, concordance between the biomarkers showed a higher trend for the progressed compared with the non‐progressed samples. Concordance in biomarker expression for non‐progressed versus progressed for ER was 64% versus 89%, for PR 47% versus 91%, for HER2 82% versus 87%, for Ki67 53% versus 60%, and for p53 67% versus 83%, respectively. To evaluate whether the differences in biomarker concordance reached statistical significance when comparing progressed with non‐progressed, we compared the frequency of biomarker concordance by Fisher's exact test. This analysis showed no significant differences in the frequency of biomarker concordance in ER, HER2, Ki67, or P53 (Fisher's exact test; *p* > 0.05). However, concordance in PR expression was significantly higher in the progressed samples than in the non‐progressed samples (Fisher's exact test; *p* = 0.027).

Furthermore, logistic regression was used to analyze whether the elevated expression of any of the clinically relevant biomarkers in patient DCIS predicated invasive progression in the MIND model. This analysis showed that among the biomarkers, only % PR expression predicted xenografted DCIS invasive progression (Table 1). Table 1 indicates the odds ratios, 95% confidence intervals, and Wald *P* values for the full model and for the reduced model after eliminating non‐significant explanatory variables.

**Table 1:** Logistic regression analysis for estimating patient biomarkers that predicted DCIS invasive progression

Full model			Final model*	
Predictor	OR (95% CI)	*P* value	OR (95% CI)	*P* value
ER	1.05 (0.99–1.22)	0.28		
**PR**	**1.03 (1.00–1.07)**	**0.048**	**1.04 (1.01–1.07)**	**0.017**
HER2	0.92 (0.34–2.65)	0.87		
Ki67	1.00 (0.91–1.12)	0.93		
P53	1.01 (0.89–1.30)	0.87		

* Predictors were treated as continuous where the units are % positive cells, and the *P* values are from Wald tests on the coefficients. One case was omitted in the multivariable analysis due to missing data (HER2 and P53) and was included in the final model.

The significant values (*P* value < 0.05) are indicated in bold.

### Patterns of cancer‐related gene mutations in patient DCISs and their corresponding xenografts

We utilized a high‐depth DNA‐targeted sequencing platform of 201 cancer‐related genes to evaluate genomic aberrations that were shared between patient DCIS lesions and their corresponding xenografts [15]. The 201 genes were selected based on their biological relevance, including mutational data in the Catalogue of Somatic Mutations in Cancer (COSMIC) and the Cancer Genome Atlas [15, 22]. The genes were found to be mutated in 5% or more of the samples across all cancer types and in 3% or more of breast cancers. DNA sequencing results were analyzed for five patient/xenograft (P/X) pairs, sample IDs 19, 33, 20, 23, and 16 (supplementary material Figure S3). Patient and xenograft information can be found in supplementary material, Table S2. For these five P/X pairs, we were able to extract sufficient DNA by laser capture microdissection (LCM) from both patient and xenograft FFPE sections for targeted sequencing. Notably, all five patient‐derived DCIS xenografts showed invasive progression. Mutation analysis was performed to compare driver mutations between each matched patient and its corresponding xenograft. Supplementary material, Table S4 lists the P/X shared variants, their associated pathways, and their relevance to human cancers. Supplementary material, Table S5 includes other relevant information, including chromosomal locations, mutated codons and amino acids, as well as their COSMIC reference numbers.

Molecular aberrations that were shared in at least one P/X pair and that were previously recognized to have deleterious effects in human cancers included *CHEK2* [K416E; 415S (SNP)] [23, 24], *EGFR* (158N) [25], *ATM* (P1054R) [26], *STK11* (D194N) [27], *PIK3CA* (E545K) [28], *KIT* (798I; M541L) [29, 30], and *RUNX1* (L56S) [31]. Notably, the listed mutations carried pathogenic scores above 0.7 [32, 33, 34], which are considered deleterious mutations. The pathogenic score is assigned using a machine learning approach (called FATHMM‐MKL) that utilizes functional annotations from the Encyclopedia of DNA Elements (ENCODE) and nucleotide‐based sequence conservation measures to predict functional consequences of both coding and noncoding sequence variants [34]. While the role of some of the molecular aberrations is currently unknown, they may still be detrimental, as the T200 gene panel included only genes with relevance to cancer.

Another notable finding in our study was that individual P/X pairs carried a unique set of deleterious mutations. For example, patient/xenograft (P/X) 19 carried a pathogenic mutation in *ATM*, P/X 33 in *EGFR* and *STK11*, P/X 20 in *EGFR* and *PIK3CA*, and P/X 16 in *RUNX1* and *KIT*. The existence of P/X unique mutations is consistent with inter‐tumoral heterogeneity and the existence of individualized drivers of DCIS malignancy. Interestingly, molecular aberrations in *CHEK2*, *EGFR*, *ATM*, *KIT*, and *RUNX1* were also found in adjacent normal cells. The detection of these aberrations in adjacent normal tissues supports a field cancerization phenomenon in which genetic alterations in adjacent normal tissues may predispose to future DCIS recurrences (supplementary material, Table S5).

In addition to finding some shared mutations, a number of private mutations were found in P/X pairs. Although this was unexpected, these findings demonstrated that our xenograft models may represent clonal subpopulations of the original patient DCIS. Another possibility is that the PDX DCIS lesions continue to evolve within the microenvironment of MIND mammary glands.

### Progressed and non‐progressed DCISs carried a similar frequency of cancer‐related mutations

**Figure 6:**
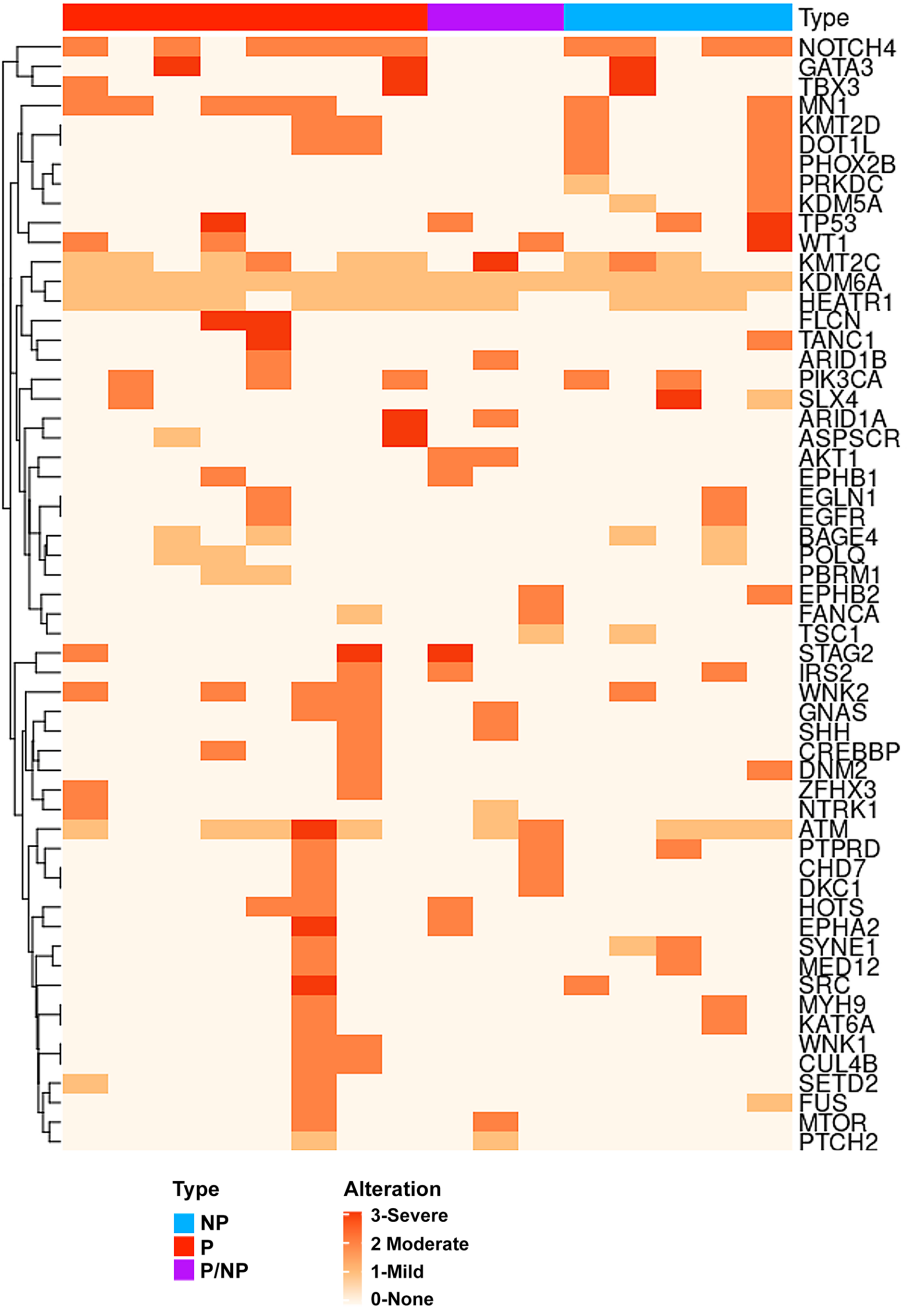
Heatmap of cancer‐related gene mutations and their severity in patient DCIS. Tempus XT oncology assay results on patient DCISs, comparing those that advanced to invasive lesions with those that remained non‐invasive in the MIND models. NP = non‐progressed; P = progressed; P/NP = mixed or both progressed and non‐progressed. Alterations are color‐coded as severe (3), moderate (2), mild (1), and none (0).

A major goal of this study was to compare mutation signatures in patient DCIS, i.e. comparing those that showed invasive progression with those that remained non‐invasive in the MIND models. The Tempus XT oncology assay, which combines a 648‐gene panel to detect clinically actionable variants from FFPE tissues, was utilized. This assay has demonstrated a high sensitivity (>95%) and specificity (>99%) for DNA‐derived variants and can identify actionable variants of both somatic and germline origins. We utilized FFPE sections of DCIS biopsy samples from 16 patients, 11 of which showed invasive progression in our MIND models, while five remained non‐invasive. Among the 11 samples, three showed a mixed pattern in which some xenografts showed invasive progression while some remained non‐invasive. A heatmap which includes patient‐specific gene variants is shown in Figure 6. The list of patient‐specific gene variants, their severity, transcript ID, and protein and cDNA changes as well as COSMIC IDs are shown in supplementary material, Table S6. The severity of the gene variants was color‐coded (Figure 6). SnpSift annotated and predicted the severity of SNPs based on their effect on gene expression and function. The variant calls include synonymous versus non‐synonymous SNPs, start codon gains or losses, stop codon gains or losses, and their genomic location such as intronic, 5′‐UTR or 3′‐UTR. Analysis of the frequency of cancer‐related pathogenic mutations among the groups showed no significant differences (P = 27, mixed = 43, NP = 79; Kruskal–Wallis: *p* ≥ 0.05). There were also no differences in the frequency of high, moderate, or mild severity mutations (P = 25 high severity, 120 moderate severity, and 50 mild severity; NP = 9 high severity, 58 moderate severity, and 28 mild severity; mixed = 3 high severity, 33 moderate severity, and 14 mild severity; Kruskal–Wallis; *p* > 0.05).

Among the groups, the most common high severity sequence variants that were also pathogenic included *ATM* (p.ARG1575His) in the progressed group, *KMT2C* (p.Ala976Thr) in the mixed group, and *SLX4* (p.Gly1447Ser) in the non‐progressed group. The most common moderate severity variants that were also pathogenic included *WNK2* (p.Arg543Trp) and *PIK3CA* (p.Glu545Lys; p.His1045Arg) in the progressed group, *AKT1* (p.Glu17Lys) and *ATM* (p.Arg1575His) in the mixed group, and *KMT2D* (p.Val3446Leu) and *PIK3CA* (p.His1047Arg; p.Glu545Lys) in the non‐progressed group. The most common mild severity variants that were also pathogenic included *KMT2C* (p.Ala976Thr) and *ATM* (p.Arg1575His) in the progressed group, *ATM* (p.Arg1575His) in the mixed group, and *ATM* (p. Arg1575His) and *KMT2C* (p.Ala976Thr) in the non‐progressed group. These results indicate that high severity and pathogenic variants existed in the patient's DCIS regardless of whether they advanced to invasive lesions or remained non‐invasive in the MIND models.

As shown in Figure 4, the invasive DCIS MIND models showed a significantly higher extent of *in vivo* growth compared with non‐invasive models. To show the contribution of specific gene mutations in patient DCIS to extent of *in vivo* growth in the DCIS MIND models, specific pathogenic mutations were listed next to each line representing median extent of DCIS MIND *in vivo* growth and IQR. As shown in supplementary material, Figure S4, pathogenic mutations existed equally in the samples regardless of their progression status. These data again support the hypothesis that cancer‐related gene mutations may not be the primary drivers of invasive DCIS.

## Discussion

Efforts to develop animal models of preinvasive breast lesions date back to 1975, when Outzen *et al* reported the transplantation of small fragments of human ‘cystic hyperplasias’ into cleared mammary fat pads of nude mice [35]. Later, in 1997, Holland *et al* reported the transplantation of fragments from 25 cases of human DCIS into athymic Balb/c nu/nu mice [36] that maintained their DCIS characteristics for up to 8 weeks. Miller and co‐workers developed MCF10AT xenografts that recapitulated the full spectrum of human breast lesions, including normal ducts, usual ductal hyperplasia, atypical hyperplasia, carcinoma *in situ*, and invasive cancers [37, 38]. A clonal derivative of MCF10AT xenografts, referred to as MCF10DCIS.com, produced comedo DCIS (a more aggressive type of DCIS with central necrosis) when transplanted at early passages into cleared fat pads of immunodeficient mice. Another premalignant cell line model, SUM225CWN, was derived from a chest wall recurrence of a ductal carcinoma lesion [39]. Similar to those of MCF10DCIS.com, xenografts of the SUM225CWN cell line formed comedo DCIS in NOD‐SCID mice [40]. Recently, Espina *et al* demonstrated the successful xenotransplantation of freshly procured DCIS organoids derived from patient DCIS biopsies or surgical specimens into cleared mammary fat pads of immunocompromised mice. Tumors formed in the mice at a rate of ~80%, and most progressed to invasive lesions [41]. Despite these efforts, none of the previous models mimicked the natural evolution of human DCIS by simulating the initial stages of DCIS intraductal growth and/or used patient‐derived cells.

With the idea that human DCIS is initiated inside the ducts, we established the MIND method in which patient‐derived DCIS surgical and biopsy specimens are digested into single epithelial cells followed by their intraductal injection [13]. This is the first patient‐derived xenograft model that recapitulates the natural evolution of human DCIS, as the cancer cells initially form *in situ* lesions inside the mammary ducts followed by their invasion as they bypass the natural barriers of the ductal myoepithelial cell layer and basement membrane. The take rate for the MIND method using primary human cells, including hyperplastic, *in situ*, and invasive epithelial cells, is 70–90%. DCIS‐like lesions injected using the MIND method mimic all histologic subtypes of human DCIS, including micropapillary, papillary, solid, comedo, and cribriform. This is remarkable because DCIS epithelial cells transplanted as single cells grow and re‐establish the various histologic subtypes. Most importantly, a fraction of xenografted DCISs undergo invasive progression, while the remainder persist as non‐invasive and non‐progressed DCIS‐like lesions. Among the 37 patient samples injected by the MIND method, 20 samples (54%) showed *in vivo* invasive progression, while 17 (45%) of the samples remained non‐invasive. The rate of invasive progression in our models is similar to the rate at which human untreated high‐grade DCIS exhibited progression to invasive breast cancer (54% MIND models versus 48% untreated human DCIS). Notably, the majority of the DCIS samples that we receive are high grade (70%), while the remaining are intermediate grade (27%) and low grade (3%). One important caveat is that DCIS progression was followed for a median of 9 months in our models, whereas human untreated DCISs were followed for a median of 3.75 years (range 1–12 years). Another important consideration is that our models are immunocompromised, and the contribution of the immune system to DCIS invasive progression is not taken into account.

A critical question in the field is whether any DCIS biomarkers can predict the risk of disease recurrence or evolution to invasive cancer. A number of previous studies have proposed biomarkers such as tumor size, nuclear grade, the presence of necrosis and expression of certain biomarkers such as HER2 overexpression, high proliferation (as detected using Ki67), p53 expression, and hormone receptor negativity in DCIS as risk factors for breast cancer recurrence [42, 43]. However, the true prognostic value of these biomarkers has been questioned since most studies suffered from patient and treatment variability, such as the extent of surgery and the use of radiotherapy or endocrine therapy [44]. An advantage of our model is that the DCIS samples have not been treated prior to their intraductal transplantation, thus removing treatment influences. Analysis of our data pointed to PR expression level in patient DCIS as the only patient‐specific biomarker that predicted invasive progression in our xenograft models. PR is one of the seven cancer‐related genes in the Oncotype DCIS score which predicts 10‐year risk of local recurrence (DCIS or invasive) and invasive local recurrence following treatment by breast conservation surgery [45]. Furthermore, progesterone and progesterone receptor (PR) are considered potent mitogens for normal and cancerous breast epithelial cells. In normal breast, progesterone/PR‐B induces the expression of WNT4, cyclin D1, and RANKL, which cause the expansion of the hormone receptor (HR)‐negative mammary epithelial cells during pregnancy. In contrast to normal human epithelial cells, HR‐positive breast tumors proliferate via autocrine mechanisms that involve PR‐induced expression of RANKL, Wnt4, and cyclin D1, as well as PR‐induced activation of protein kinases including CDK2, c‐Src, CK2, MAPK, and PI3K/AKT [46]. Large clinical studies have demonstrated that progestin added to hormone replacement therapy significantly increased the incidence and grade of breast cancers in post‐menopausal women, while there was no increased risk associated with estrogen alone [47]. Furthermore, estrogen‐only hormone replacement therapy was protective in some women [48]. Recent studies have also demonstrated that progesterone and PR promote the expansion and self‐renewal of breast stem and progenitor cells [49]. Since mammary stem cells may be the primary targets of carcinogenic transformation, it is postulated that progesterone/PR may likely also induce the expansion of cancer stem cells, resulting in breast cancer progression and/or recurrence. These studies, as well as our data, point to the potential therapeutic benefit of progesterone inhibition for prevention of DCIS malignancy. Thus, the finding that PR was the only biomarker that predicted DCIS MIND xenograft invasive progression further validates our model as a tool for identifying biomarkers of aggressive DCIS and potential molecular underlying mechanisms of DCIS with invasive potential.

Another important finding of this study was that the invasive DCIS MIND models showed significantly higher median extent of *in vivo* growth compared with non‐invasive and mixed invasive and non‐invasive xenografts. Tumor size has been proposed as a biomarker of aggressiveness in DCIS and this finding also validated our model as a valuable tool for studying the underlying mechanisms of DCIS invasive progression. Notably, there was a significant level of inter‐ and intra‐tumoral heterogeneity with respect to the xenografts' extent of *in vivo* growth and invasiveness. Importantly, nine samples showed a mixed pattern in which some xenografted DCISs advanced to invasive lesions while others remained non‐invasive. These data indicate that both invasive and non‐invasive cells co‐existed within a single DCIS sample and again highlight DCIS cellular heterogeneity, similar to other reports [50, 51].

A side‐by‐side comparison of patient and xenografted DCIS biomarkers (ER, PR, HER2, Ki67, and p53) showed lack of 100% concordance. While this was unexpected, due to cellular heterogeneity, a single section from a xenograft and patient DCIS used for biomarker studies may not represent the entire DCIS clonal cell subpopulations. Nonetheless, biomarker expression showed higher concordance in the xenografts that exhibited invasive progression compared with xenografts that remained non‐invasive.

To determine whether the mutation landscape in xenografts mimicked that of the corresponding patient DCIS, we performed high‐depth targeted sequencing of DNA isolated from five pairs of patient DCISs and their corresponding xenografts [15]. While all five P/X pairs shared mutations in *TP53*, *PDGFRA*, *KMT2C*, *CHECK2*, and *EGFR*, there were also P/X pair unique pathogenic mutations, including *ATM* (P/X 19), *STK11* (P/X 33), *PIK3CA/KIT* (P/X 20), *NOTCH1* (P/X 23), and *RUNX1/KIT* (P/X 16). There were also a number of private mutations in patient DCISs and their corresponding xenografts. The existence of private mutations in xenografts supports DCIS multi‐clonality. Others previously reported the potential existence of multiple subclones within DCIS. For example, Allred *et al* reported that a large percentage of DCIS lesions (48%) exhibited intralesional heterogeneity [50]. Intralesional heterogeneity reflects regions within a single DCIS lesion exhibiting different nuclear grades, histologic grades, and degrees of biomarker expression [50]. Park *et al*, using immunofluorescence *in situ* hybridization (IFISH) for common genomic aberrations in breast cancer (i.e. 8q24), also showed a high degree of genetic heterogeneity among cells within each patient's DCIS [52]. Recently, cellular barcoding and single‐cell genomic sequencing have also demonstrated the existence of multiple clones within invasive breast cancers as well as DCIS [53, 54, 55]. Therefore, we hypothesize that the MIND models mimic clonal heterogeneity of human DCIS and that the xenografts may represent clonal subpopulations of the patient sample from which they were derived.

We also analyzed the mutation signature of patient DCIS by using the Tempus XT oncology assay on 16 archival patient DCIS samples, 11 of which showed invasive progression in our MIND models, while five remained non‐invasive. Among the 11 patient samples, three showed a mixed pattern in which some xenografts showed invasive progression, while others remained non‐invasive. This analysis showed that there were no significant differences in the type, number, or severity of mutations among the groups. Furthermore, there was no significant correlation between the type, number or severity of mutations and the extent of xenografted DCIS *in vivo* growth. These data are in agreement with one previous study which showed no significant differences in the frequency of non‐synonymous mutations and CNAs when comparing pure DCIS with synchronous IDC to DCIS that did not progress to invasion for a median follow‐up of 72 months (range 22–85 months) [51]. While there were numerically higher numbers of mutations in key cancer genes such as *TP53* and *PIK3CA* in DCIS that progressed to invasion, the results did not reach statistically significant differences. In addition, the repertoires of somatic mutations were significantly different according to ER and HER2 status [51]. One caveat of our study is that the majority of our samples were ER/PR‐positive and intermediate to high grade. Therefore, the lack of finding significant differences in mutational signature among the groups could be due to small sample size.

One potential limitation of our mouse models is the lack of human microenvironments, including the immune cells and stroma. Studies comparing immune microenvironments in DCIS, DCIS with associated IDC, and IDC have reported the existence of a more immunosuppressive environment associated with a transition from DCIS to IDC [56]. DCIS also contained a more clonally expanded population of T cells than did IDC, supporting the idea that DCIS cells with an invasive potential may induce immunosuppression upon invasion into the stroma [56]. Other studies have found a positive correlation between tumor‐infiltrating lymphocytes (TILs) and histological features of poor DCIS prognosis including high nuclear grade, comedo necrosis, and HR‐negative and high Ki67 DCIS [57]. While an association between TILs and DCIS recurrence was not found, that study did find higher TIL infiltration to be associated with telomeric imbalances and *TP53* mutations. A recent study found higher FOXP3^+^ TILs to be associated with DCIS that showed a future recurrence in six cases [58]. Therefore, inter‐individual heterogeneity in DCIS epithelial cells may result in the differential recruitment of a subset of immune cells, i.e. immunosuppressive TILs, which in turn influence DCIS invasive progression and/or recurrence. Since our DCIS MIND animal models are currently immunocompromised, these interesting aspects of DCIS biology will need to be addressed in future studies.

In conclusion, MIND is a valuable tool for studying the molecular and cellular mechanism underlying DCIS invasive progression. Since our studies showed a significant level of DCIS inter‐ and intra‐tumoral heterogeneity with respect to invasive progression, extent of *in vivo* growth, and mutation landscape, the molecular drivers of DCIS malignancy may be specific to each DCIS lesion. Furthermore, since our studies showed that genetic changes were not the primary drivers of DCIS malignancy, future studies should focus on studying other factors including clonal heterogeneity, stroma, and epigenomic reprogramming. These future studies will help to ultimately achieve the goal of identifying more reliable indicators for dangerous lesions and more effective treatments.

## Author contributions statement

YH, DL, HSE, HH, HH, HH, MR, CK and EW collected the data, performed analyses, and contributed to manuscript write‐up. MX and JZ contributed data and data analysis tools. LM, TC, MI, MR, JG, OW, AA, AH, CB, JLW, ALM, KEL, LR, OT, HR, ROM and RM contributed tissue, conceived and designed the analysis, and contributed to manuscript write‐up. JT and SGH contributed to data statistical analysis. AKG, AF, AT, ESH and FF conceived and designed the analysis, and contributed to manuscript write‐up. FB conceived and designed the analysis and wrote the manuscript. All the authors reviewed and approved the manuscript.

## Supporting information


**Figure S1.** Rate of engraftment in the MIND model following intraductal transplantation of human breast malignant and nonmalignant epithelial cells
**Figure S2.** Representative IF images of non‐progressed and progressed PDX DCIS MIND models
**Figure S3.** T200 targeted sequencing‐DNA mutational analysis between primary patient DCISs lesions and corresponding xenografts
**Figure S4.** Contribution of pathogenic mutations to PDX DCIS MIND *in vivo* growth and progression

**Table S1.** List of antibodies used

**Table S2.** List of patients and their corresponding xenografts, progression status, extent of xenograft growth and IQR, patient and xenograft histology and biomarkers, and patient demographics

**Table S3.** Comparison of patients' and their corresponding xenografts' biomarker expression

**Table S4.** Molecular aberrations shared in patients and their corresponding xenografts

**Table S5.** T200 targeted sequencing analyzed data

**Table S6.** Tempus XT targeted sequencing analyzed data

## Data Availability

Analyzed T200 and Tempus XT targeted sequencing data have been provided as supplementary material, Tables S5 and S6. Raw data will be provided upon request by FB.
