# Supplementary material for: Mouse‐INtraDuctal (MIND): an in vivo model for studying the underlying mechanisms of DCIS malignancy
Source: J Pathol. 2021 Dec 13;256(2):186–201. doi: 10.1002/path.5820 (PMC8738143; doi:10.1002/path.5820)
Supplement: Supplementary file 1 — Figure S1. Rate of engraftment in the MIND model following intraductal transplantation of human breast malignant and nonmalignant epithelial cells Figure S2. Representative IF images of non‐progressed and progressed PDX DCIS MIND models Figure S3. T200 targeted sequencing‐DNA mutational analysis between primary patient DCISs lesions and corresponding xenografts Figure S4. Contribution of pathogenic mutations to PDX DCIS MIND in vivo growth and progression [file PATH-256-186-s003.docx]

**Mouse-INtraDuctal (MIND): an *in vivo* model for studying the underlying mechanisms of DCIS malignancy**

Y Hong *et al. J Pathol* DOI: 10.1002/path.5820

**Supplementary Figures S1–S4**

**
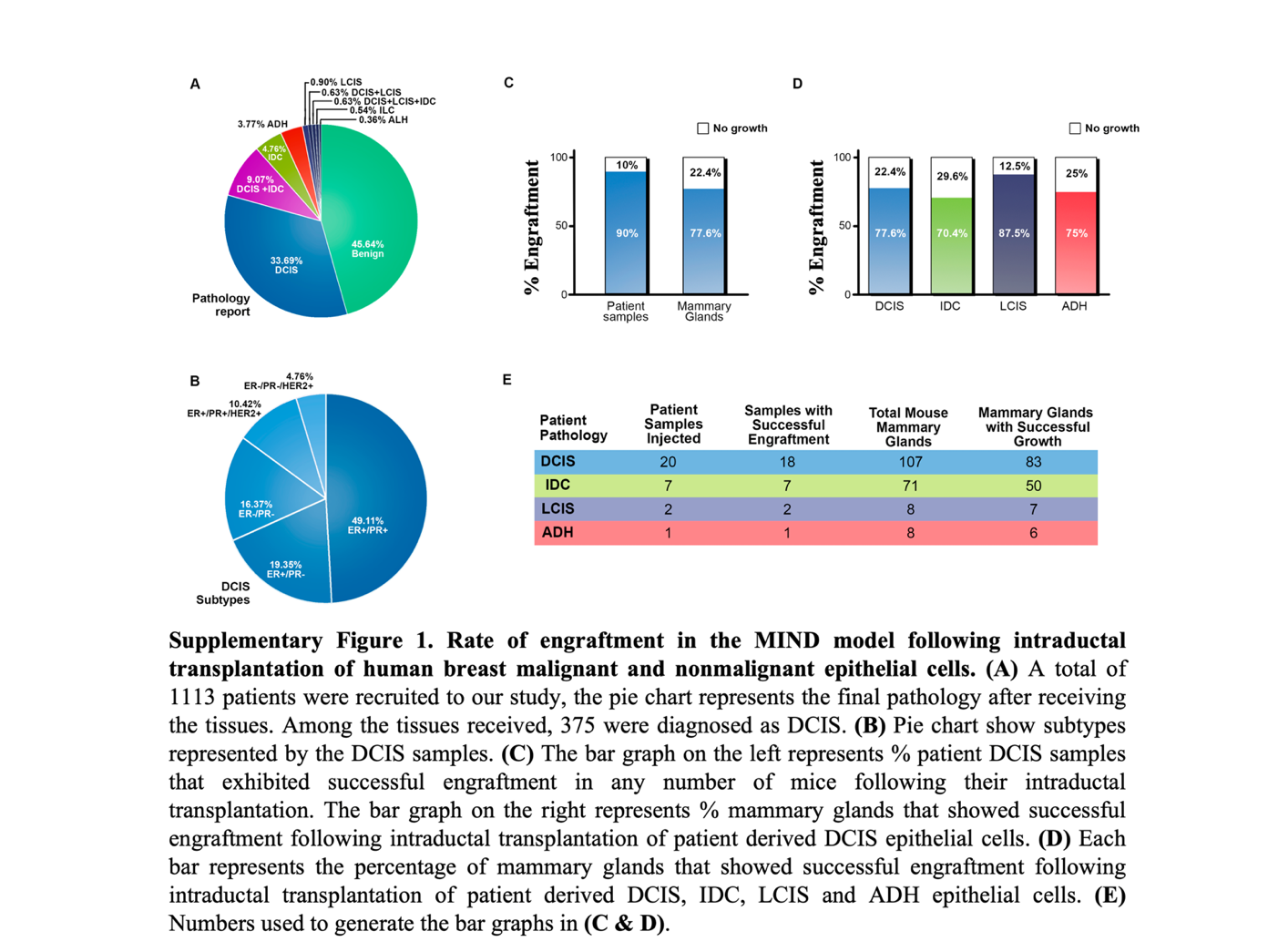
**

**Figure S1. Rate of engraftment in the MIND model following intraductal transplantation of human breast malignant and nonmalignant epithelial cells.** (A) A total of 1113 patients were recruited to our study. The pie chart presents the final pathology after receiving the tissues. Among the tissues received, 375 were diagnosed as DCIS. (B) Pie chart shows subtypes present in the DCIS samples. (C) The bar chart on the left presents the proportion of patient DCIS samples that exhibited successful engraftment in any number of mice following intraductal transplantation. The bar chart on the right presents the proportion of mammary glands that showed successful engraftment following intraductal transplantation of patient-derived DCIS epithelial cells. (D) Each bar represents the percentage of mammary glands that showed successful engraftment following intraductal transplantation of patient-derived DCIS, IDC, LCIS, and ADH epithelial cells. (E) Numbers used to generate the bar graphs in C, D.


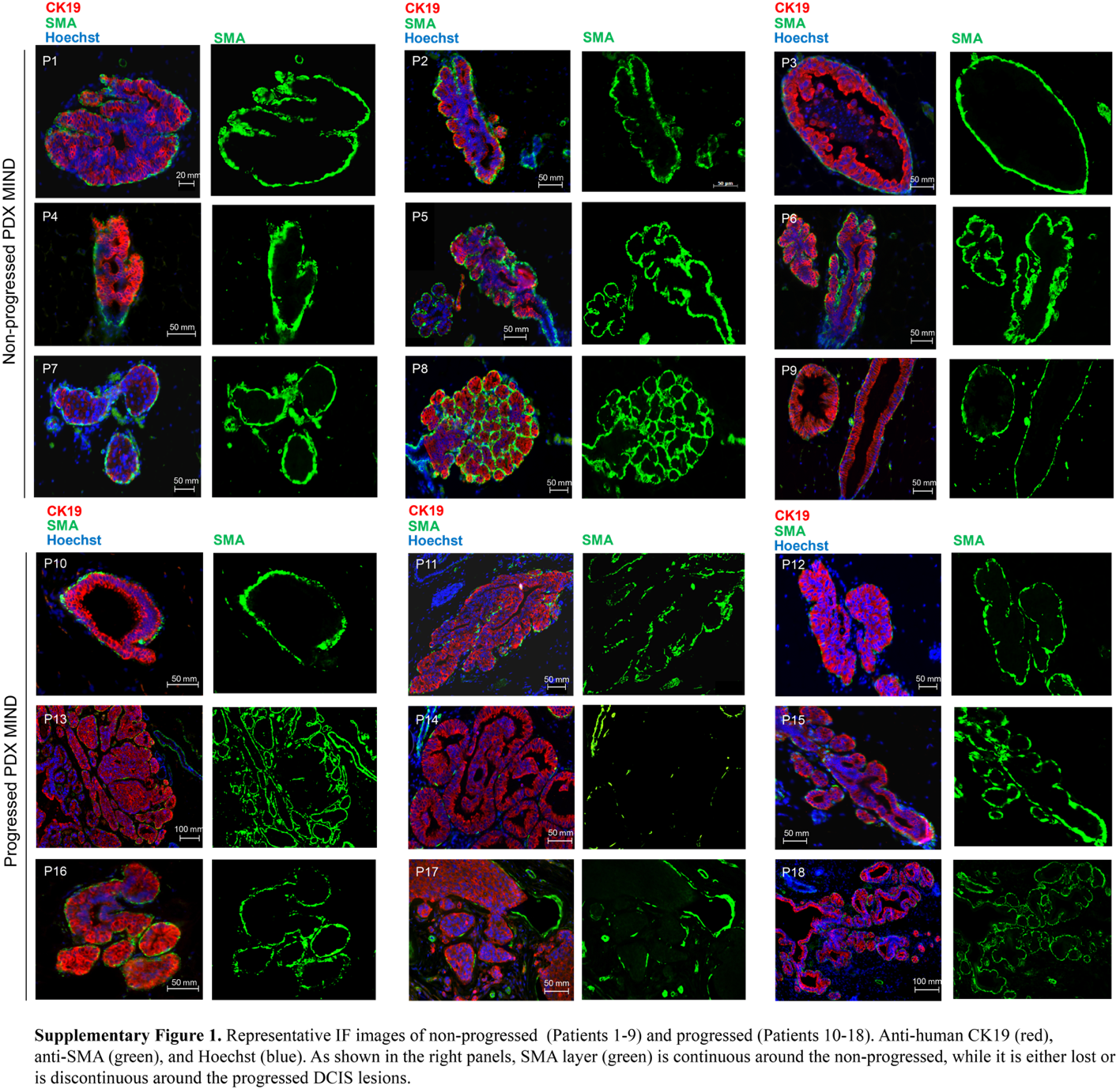


**Figure S2. Representative IF images of non-progressed and progressed PDX DCIS MIND models.** The top panel shows IF images of non-progressed (patients 1–8, 12) and the lower panel progressed (patients 16, 17, 19–23, 25–26) PDX DCIS MIND models. The panels to the right of each image include SMA only (green) demonstrating a continuous layer around the non-progressed (top panel) and either a lost or discontinuous SMA layer around the progressed models. Anti-human CK19 (red), anti-SMA (green), and Hoechst (blue).


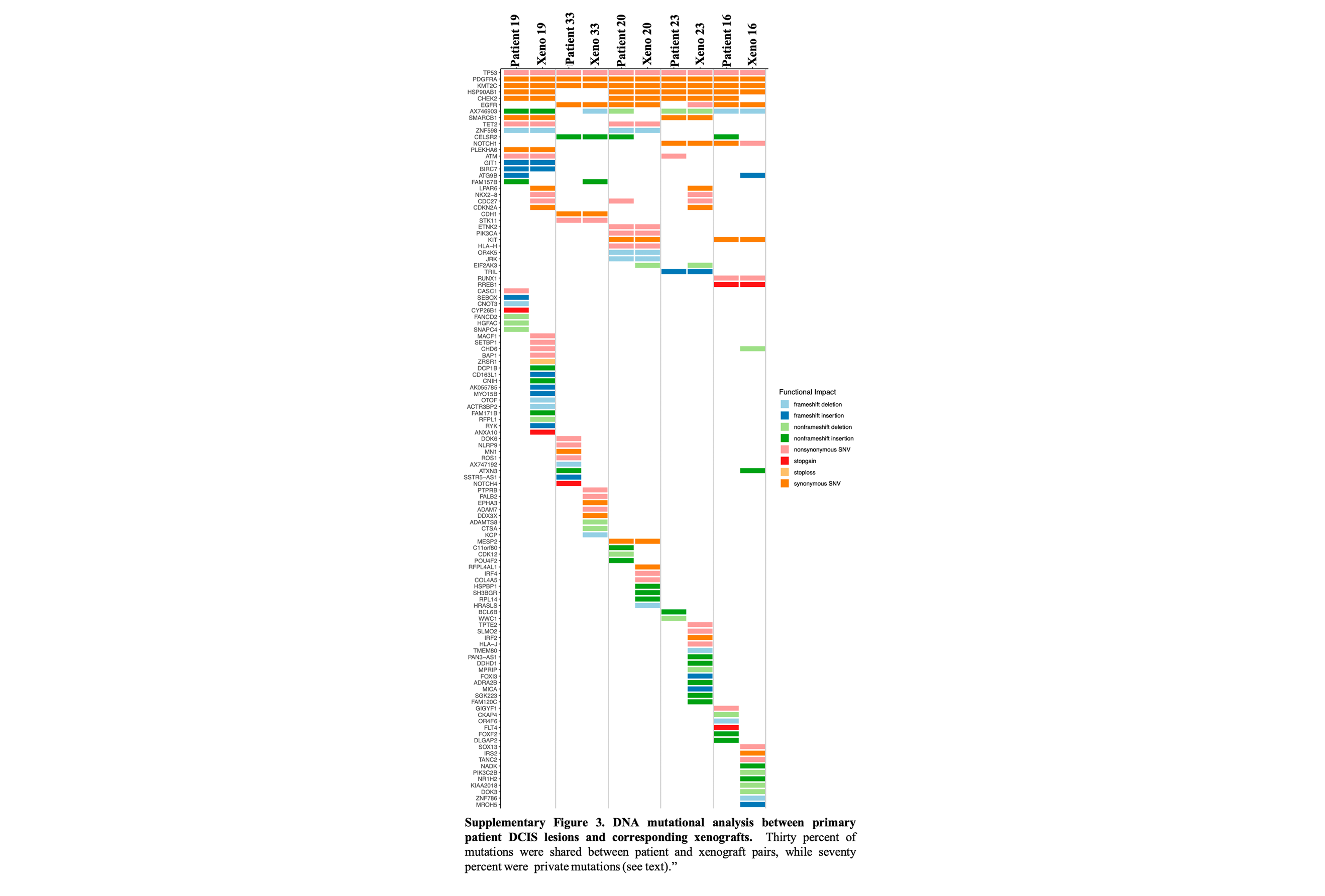


**Figure S3. T200 targeted sequencing-DNA mutational analysis between primary patient DCISs lesions and corresponding xenografts.** Thirty percent of mutations were shared between patient and xenograft pairs, while seventy percent were private mutations (see main text).

A


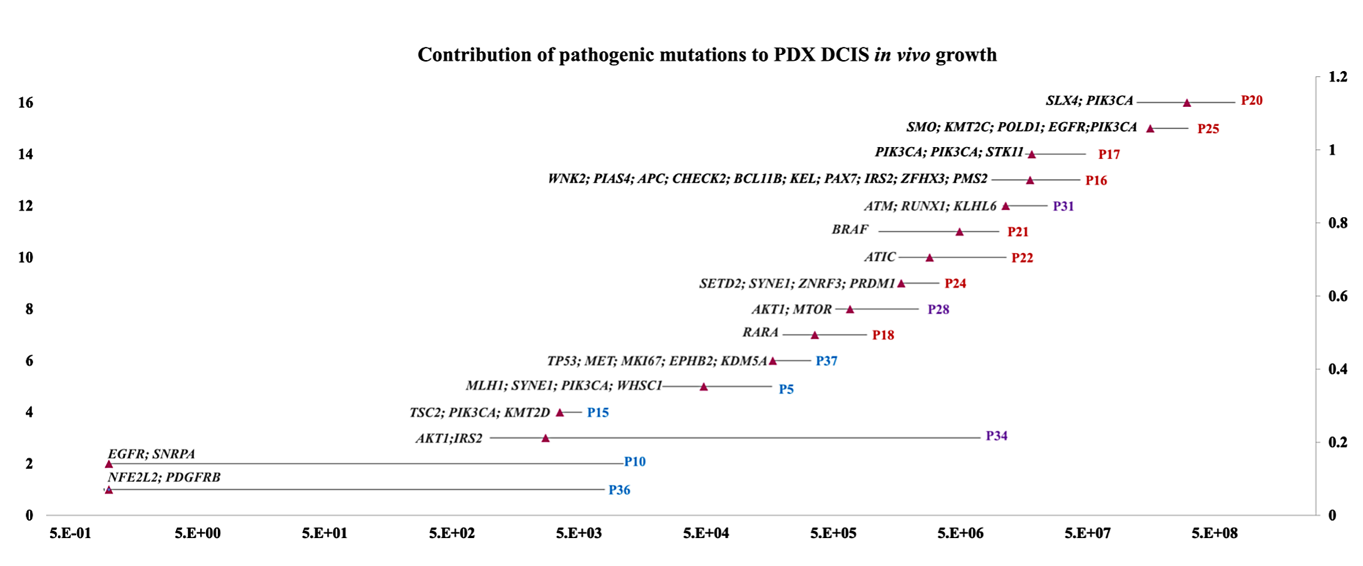


B

**
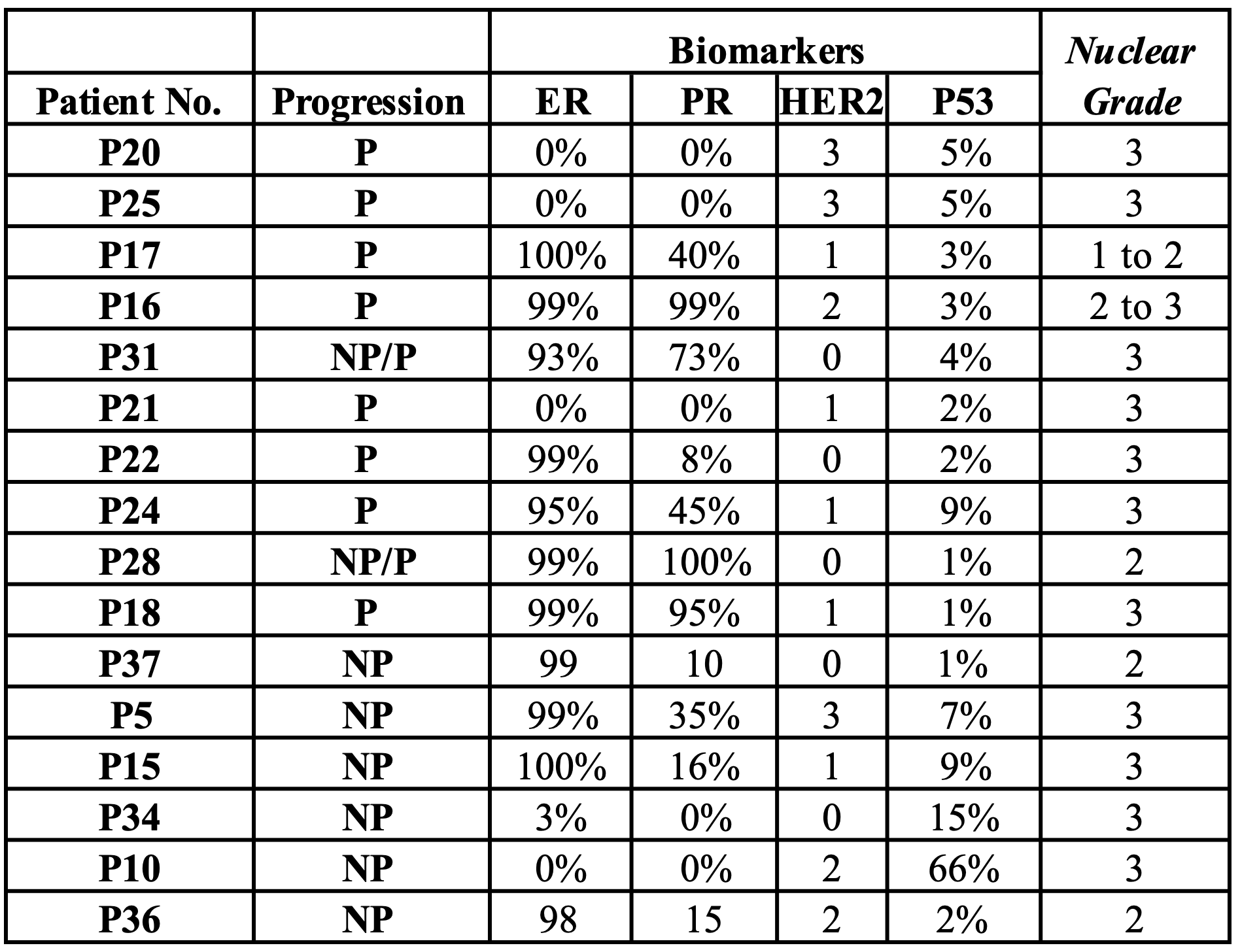
**

**Figure S4. Contribution of pathogenic mutations to PDX DCIS MIND *in vivo* growth and progression.** (A) Patient DCIS specific pathogenic mutations are listed next to each line that represents PDX DCIS MIND extent of *in vivo* growth and IQR. (B) List of patient IDs, progression status, biomarkers, and nuclear grades.
