# Supplementary material for: Mouse‐INtraDuctal (MIND): an in vivo model for studying the underlying mechanisms of DCIS malignancy
Source: J Pathol. 2021 Dec 13;256(2):186–201. doi: 10.1002/path.5820 (PMC8738143; doi:10.1002/path.5820)
Supplement: Supplementary file 2 — Table S1. List of antibodies used [file PATH-256-186-s006.docx]

**Mouse-INtraDuctal (MIND): an *in vivo* model for studying the underlying mechanisms of DCIS malignancy**

Y Hong *et al. J Pathol* DOI: 10.1002/path.5820

**Table S1.** List of antibodies used

|  | **Company** | **Catalog #** | **Dilution** |
| --- | --- | --- | --- |
| **IF staining** |  |  |  |
| Primary antibodies |  |  |  |
| Cytokeratin 5 | Vector Laboratories Inc, Burlingame, CA, USA | VPC400 | 1:25 |
| Cytokeratin 19 | Thermo Fisher Scientific, Waltham, MA, USA | MS198 | 1:50 |
| SMA | Thermo Fisher Scientific | PA5-18292 | 1:50 |
| Secondary antibodies |  |  |  |
| Alexa Fluor 488 anti-rabbit | Invitrogen, Grand Island, NY, USA | A11008 | 1:200 |
| Alexa Fluor 594 anti-mouse | Invitrogen | A21203 | 1:200 |
| Alexa Fluor 488 anti-mouse | Invitrogen | A11012 | 1:200 |
|  |  |  |  |
| **IHC staining** | | | |
| ER | Dako, Carpinteria, CA, USA | SP1 |  |
| PR | Dako | PgR 636 |  |
| HER2 | Dako Hercep test | Per FDA | Per FDA |
| Ki67 | Dako | MIB-1 |  |
| P53 | Dako | DO-7 |  |
| Cytokeratin5/6 | Biocare Medical, Pacheco, CA, USA | CM 105 A, B, C | 1:200 |
| Cytokeratin 19 | Biocare Medical | CM 242 A, C | 1:50 |
| CD31 |  |  |  |
| **Magnetic sorting** |  |  |  |
| Mouse MHC I | BD Pharmingen, Franklin Lakes, NJ, USA | 553546 | 1:100 |
| Mouse MHC II | BD Pharmingen | 553564 | 1:100 |
| **Flow cytometry** |  |  |  |
| Human EpCAM | Abcam, Cambridge, MA, USA | ab37305 | 1:20 |
