# Supplementary material for: Mouse‐INtraDuctal (MIND): an in vivo model for studying the underlying mechanisms of DCIS malignancy
Source: J Pathol. 2021 Dec 13;256(2):186–201. doi: 10.1002/path.5820 (PMC8738143; doi:10.1002/path.5820)
Supplement: Supplementary file 5 — Table S4. Molecular aberrations shared in patients and their corresponding xenografts [file PATH-256-186-s004.docx]

**Mouse-INtraDuctal (MIND): an *in vivo* model for studying the underlying mechanisms of DCIS malignancy**

Y Hong *et al. J Pathol* DOI: 10.1002/path.5820

**Table S4.** Molecular aberrations shared in patients and their corresponding xenografts

Reference numbers refer to the main text list

| **Molecular alterations** | **Patient DCIS/xenograft pairs** | **Mechanism** | ***Pathway[22,24]** | **References** |
| --- | --- | --- | --- | --- |
| *TP53_P87R* | 19,33,20,23,16 | Tumor suppressor and most commonly mutated gene in human cancers |  | [59] |
| *PDGFRA_P567P; PDGFRA_V824V (pt/xeno 5 only)* | 19,33,20,23,16 | High expression has been associated with aggressive breast cancers, e.g. the TNBC subtype, and increased the risk of 5-year distant recurrence. PDGFRα was significantly upregulated in lymph node metastases |  | [60] |
| KMT2C_S806S | 19,33,20,23,16 | MLL3; tumor suppressor. Frequently mutated in aggressive forms of glioblastoma, pancreatic cancers and melanoma. KMT2C deletion in breast cancer was associated with significantly shorter progression-free survival. Cells with loss of KMT2C activity are deficient in homologous recombination‐mediated double‐strand break DNA repair and suffer from DNA damage and genomic instability. The cells rely on PARP1/2 for DNA repair, suggesting that cancer cells with low KMT2C expression may respond favorably to PARP1/2 inhibition therapy |  | [61–63] |
| HSP90AB1_G713G | 19,20,23,16 | HSP90AB1 upregulation is associated with increased risk for recurrence in TNBC and poor prognosis in ER^+^/HER2^−^ breast cancer |  | [64] |
| CHEK2_K416E and CHEK2_S415S | 19,20,23,16 | Pathogenic score *CHEK2 (K416E) =* 0.77. A serine/threonine kinase implicated in DNA repair, cell cycle arrest, and apoptosis in response to DNA damage. Alterations in *CHEK2* have been associated with resistance to chemotherapy. Loss-of-function mutations have been reported in multiple types of cancers including breast | Cell cycle checkpoint, DNA damage response | [22–24] |
| EGFR_N158N | 33,20,16 | Pathogenic synonymous SNV in *EGFR* (N158) (score 0.83). Mutations in *EGFR* have been associated with higher risk of breast cancer relapse and resistance to chemotherapy | Androgen receptor signaling, integrin signaling, focal adhesion, MAPK signaling, EGFR1 signaling, regulation of actin cytoskeleton, delta-notch signaling, ErbB signaling | [25] |
| SMARCB1_S308S | 19,23 | SMARCB1 is a tumor suppressor and a component of the SWI/SNF chromatin-remodeling complex. SMARCB1 is inactivated in nearly all pediatric rhabdoid tumors. SMARCB1 inactivation and resultant mis-targeting of SWI/SNF complexes to super-enhancers played a key role in rhabdoid tumor cell survival. SMARCB1 tumor suppressor mechanism may also be related to binding to c-Myc and inhibition of its transcriptional activity |  | [65,66] |
| TET2_L1742W | 19,29 | *TET2* is an enzyme that plays a key role in DNA methylation and is mutated in multiple cancers including breast |  | [67] |
| ZNF598_G42fs | 19,20 | ZNF598 is an E3 ubiquitin ligase. The ubiquitin proteasome system (UPS) regulates diverse cellular processes by targeting specific substrate proteins for ubiquitination and degradation. Implicated in ribosome associated quality control and surveillance mechanism |  | [68] |
| NOTCH1_D1698D | 23 | Aberrant NOTCH pathway activation can cause mammary tumors in mice. One study reported that patients with tumors expressing high levels of NOTCH1 exhibited a significantly poorer overall survival. Another study of 115 tumor tissues demonstrated that NOTCH1 expression was significantly associated with higher TNM stage, metastasis, and triple-negative breast cancers |  | [69,70] |
| KIT_I798I (17) KIT_M541L (13) KIT_L862L (13) | 20,16 | This particular SNV carries a pathogenic score of 0.73. *KIT*, also known as CD117, encodes a receptor tyrosine kinase implicated in cell proliferation and migration. Studies have highlighted the role of KIT in numerous human cancers including melanoma, brain tumors, AML, and gastrointestinal stromal tumors (GIST). While the exact role of KIT in human breast cancers is controversial, one study reported that loss of KIT may be associated with the development of invasive tumors and metastasis | KIT receptor signaling pathway | [30,71] |
| ATM_P1054R | 19 | ATM carried a pathogenic score of 0.97. A germline mutation in *ATM* is associated with increased life time risk of a number of cancers including breast, lymphoid, gastric, and skin cancers. Somatic *ATM* mutations are also commonly found in a number of solid cancers including breast. *ATM* mutations have been associated with poor outcome and resistance to chemotherapy | Cell cycle checkpoint, DNA damage response (DDR), miRNAs involved in DDR, homologous recombination | [26] |
| GIT1_E631fs | 19 | G-protein-coupled receptor kinase interacting protein 1 (*GIT1*) activates Rac1/Cdc42. High expression of GIT1 is associated with a significantly lower overall and disease-free survival in patients with non-small-cell lung cancer. *In vitro* studies found GIT1 expression to increase the invasiveness of NSCLC cells by stimulating Rac1/Cdc42 |  | [72] |
| BIRC7_P223fs | 19 | *BIRC7* is a member of inhibitors of apoptosis that may play a role in the development of adrenocortical tumors through the inhibition of caspases |  | [73] |
| CELSR2_T12delins | 33 | CELSR2 (EGF-like protein 2) is part of the cadherin subfamily with increased cytoplasmic staining in breast cancer cells compared to benign epithelium, suggesting a possible role of CELSR2 in the pathogenesis of human breast cancers. Univariate and multivariate Cox regression analyses showed that CELSR2 may be an independent risk factor in hepatocellular carcinomas |  | [74,75] |
| CDH1_N751N | 33 | Abnormal E-cadherin (CDH1) expression in the primary tumors with re-expression in lymph node metastasis is a common event in breast cancer and may be an independent prognostic biomarker in predicting a shorter survival in node-positive breast cancer patients |  | [76] |
| STK11_D194N | 33 | In breast cancer cell line models, STK11 overexpression reduced tumor growth, microvessel density formation, and lung metastasis by downregulating matrix metalloproteinase-2, matrix metalloproteinase-9, vascular endothelial growth factor, and basic fibroblast growth factor. The *STK11* mutation reported here is considered pathogenic with a score of 0.97 | Integration of energy metabolism; signaling by insulin receptor, AMPK signaling, TGF-B receptor signaling pathway | [27] |
| PIK3CA_E545K | 20 | *PIK3CA* is commonly mutated in breast cancers. PIK3CA plays a key role in the control of various cellular processes including cell growth, proliferation, metabolism, and migration. Studies have shown that this pathway is upregulated in up to 70% of human cancers. *PIK3CA*-mutated breast cancers show poor outcome and resistance to chemotherapy. The specific *PIK3CA* mutation in this sample carried a pathogenic score of 0.97 | AMPK signaling, TLR signaling, angiogenesis, integrin signaling, IL-3, IL-2 and IL-4 signaling, focal adhesion, EGFR1 signaling, regulation of acting cytoskeleton, G13 signaling, estrogen signaling | [81] |
| JRK_W463fs | 20 | JRK is a newly identified oncogene. Depletion of JRK in cancers suppressed β-catenin transcriptional activity and reduced cell growth. JRK is aberrantly expressed in several cancers including colorectal, breast, and ovarian cancers. Some have proposed that JRK may serve as a potential new therapeutic target in breast cancer |  | [80] |
| RUNX1_L56S | 16 | RUNX1 is a transcription factor that regulates hematopoietic stem cell differentiation. Defects in this gene have been associated with myeloid and lymphoid leukemias. Loss of function of *RUNX1* by inactivating mutations has been associated with enhanced estrogen signaling and the development of luminal breast cancers. A 17-gene expression signature that distinguished primary tumors from metastases included RUNX1. RUNX1 loss of function has been reported to promote EMT by transcriptional inactivation of E-cadherin. The specific *RUNX1* mutation in our sample carries a pathogenic score of 0.90 |  | [31,78,79] |
| RREB1_C219X | 16 | RREB1 is a transcription factor that specifically binds to the RAS-responsive elements and promotes collective cell migration |  | [77] |
